# Cortical Presynaptic Boutons Progressively Engulf Spinules as They Mature

**DOI:** 10.1523/ENEURO.0426-19.2020

**Published:** 2020-10-14

**Authors:** Charles Campbell, Sarah Lindhartsen, Adam Knyaz, Alev Erisir, Marc Nahmani

**Affiliations:** 1 Division of Sciences and Mathematics, University of Washington | Tacoma, Tacoma, WA 98402; 2 Department of Psychology, University of Virginia, Charlottesville, VA 22904

**Keywords:** critical period, developmental plasticity, electron microscopy, focused ion beam scanning electron microscopy, presynaptic terminal, synaptic plasticity

## Abstract

Despite decades of discussion in the neuroanatomical literature, the role of the synaptic “spinule” in synaptic development and function remains elusive. Canonically, spinules are finger-like projections that emerge from postsynaptic spines and can become enveloped by presynaptic boutons. When a presynaptic bouton encapsulates a spinule in this manner, the membrane apposition between the spinule and surrounding bouton can be significantly larger than the membrane interface at the synaptic active zone. Hence, spinules may represent a mechanism for extrasynaptic neuronal communication and/or may function as structural “anchors” that increase the stability of cortical synapses. Yet despite their potential to impact synaptic function, we have little information on the percentages of developing and adult cortical bouton populations that contain spinules, the percentages of these cortical spinule-bearing boutons (SBBs) that contain spinules from distinct neuronal/glial origins, or whether the onset of activity or cortical plasticity are correlated with increased prevalence of cortical SBBs. Here, we employed 2D and 3D electron microscopy to determine the prevalence of spinules in excitatory presynaptic boutons at key developmental time points in the primary visual cortex (V1) of female and male ferrets. We find that the prevalence of SBBs in V1 increases across postnatal development, such that ∼25% of excitatory boutons in late adolescent ferret V1 contain spinules. In addition, we find that a majority of spinules within SBBs at later developmental time points emerge from postsynaptic spines and adjacent boutons/axons, suggesting that synaptic spinules may enhance synaptic stability and allow for axo-axonal communication in mature sensory cortex.

## Significance Statement

Synaptic spinules are finger-like projections from neurites that can become completely embedded within presynaptic boutons, potentially enhancing synaptic communication and stability. Yet while their existence has been discussed for decades, spinule prevalence, projection origins, and relationship to neocortical sensory activity remain unknown. In this study, we employed 2D and 3D electron microscopy techniques to characterize the development of excitatory cortical spinule-bearing boutons (SBBs) and their relationship to sensory activity and plasticity. Our results demonstrate that neocortical presynaptic SBB prevalence is not correlated with the onset of sensory activity or heightened cortical plasticity and that by late adolescence nearly one-quarter of presynaptic boutons contain a spinule. Hence, synaptic spinules may play progressively important roles in cortical function as these synapses mature.

## Introduction

Synapses underlie the sensory, motor, and cognitive processing functions of nervous systems, and alterations of synaptic communication and stability directly affect animal behavior and memory (Sherrington, 1906; Liewald et al., 2008; Liu et al., 2012). Moreover, changes in synaptic morphology can accurately predict changes in synaptic strength and stability (Murthy et al., 2001; Ostroff et al., 2002; Branco et al., 2010; Holderith et al., 2012; Araya et al., 2014; Meyer et al., 2014; Quinn et al., 2019). However, a ubiquitous and potentially crucial synaptic structure, the synaptic spinule, remains enigmatic and underexplored.

Spinules are components of synapses that are conserved across the animal kingdom (Case et al., 1972; Bailey et al., 1979) yet have an unknown function. Canonically, spinules are thin, finger-like invaginating projections from postsynaptic dendritic spines that can become enveloped by other neurites (Pappas and Purpura, 1961; Westrum and Blackstad, 1962; Tarrant and Routtenberg, 1977; Spacek and Harris, 2004). When a neuronal structure such as a presynaptic bouton envelops a spinule, the membrane apposition between the spinule-projecting structure and the enveloping bouton increases, in some cases up to 26 times the size of a synapse’s “active zone” (Rodriguez-Moreno et al., 2018). Thus, spinules may represent an undescribed mechanism for neuronal communication, and/or they may function as structural “anchors” that increase synaptic strength and stability. If true, an increase in spinule-bearing synapses within a functionally defined microcircuit could enable large changes in brain function, such as increased memory retention or improved recovery after injury.

To date, the best anatomically and physiologically characterized form of spinules are those of the mammalian hippocampus, specifically spinules emanating from the postsynaptic spines of CA1 stratum radiatum and dentate gyrus pyramidal cell dendrites in adult rat (Tarrant and Routtenberg, 1977; Geinisman et al., 1994; Toni et al., 1999; Spacek and Harris, 2004). While spinules often originate from spines and become engulfed by presynaptic boutons, the term “spinules” has broadened to describe any projection from a neurite or glia that becomes enveloped within another neuronal or glial process (Petralia et al., 2015). In CA1 stratum radiatum, ∼32% of spines project spinules, and most of these spinules are enveloped by presynaptic boutons (∼90%; Spacek and Harris, 2004). Intriguingly, the percentage of CA1 spines displaying spinules increases following increases in neuronal activity, such as during electrical and chemical long-term potentiation (LTP; Geinisman et al., 1994; Toni et al., 1999; Harris et al., 2003; Ueda and Hayashi, 2013), application of high extracellular K^+^ (Tao-Cheng et al., 2009), and estradiol receptor activation (Murphy and Andrews, 2000). These data have led to the suggestion that spinules function as activity-dependent neuronal circuit remodeling and signaling elements throughout the brain (Spacek and Harris, 2004) or as a mechanism of presynaptic membrane retrieval during periods of heightened activity (Tao-Cheng et al., 2009).

However, whether these results from hippocampus generalize to other areas of the brain (e.g. neocortex) remains unclear, and whether heightened levels of developmental activity/plasticity correlate with heightened levels of spinule prevalence in vivo remains unknown. Furthermore, few studies have investigated the percentages of presynaptic boutons that contain spinules, a key component in understanding the potential for spinule-induced synaptic communication and/or stability. For example, a 2D ultrastructural study of thalamocortical (TC) boutons found that 28% of TC boutons in layer 4 (L4) of primary visual cortex (V1) contained 2D spinule-like (i.e., putative spinule) profiles (Erisir and Dreusicke, 2005), and a 3D study of TC boutons in L4 of barrel cortex reported that 13% of reconstructed postsynaptic dendritic spines projected a spinule into TC boutons (Rodriguez-Moreno et al., 2018). Thus, spinules may be a prominent feature of neocortical synapses.

Here, we used the broader definition of spinules to describe invaginating projections from neurites or glia that are enveloped by excitatory cortical presynaptic boutons. We sought to determine the proportion of synaptic excitatory spinule-bearing boutons (SBBs) within the excitatory bouton population in V1 across postnatal development, and the origins of spinules projecting into these SBBs. In addition, we examined whether the onset of sensory activity or heightened levels of cortical plasticity correlated with a commensurate increase in the proportion of SBBs in V1. Using 2D and 3D electron microscopy analyses, we find that (1) the proportion of SBBs increases in parallel with the waning of developmental plasticity and strengthening of excitatory synapses in L4 of V1, (2) SBBs preferentially envelop spinules from postsynaptic spines and adjacent boutons/axons as they mature, and (3) ∼25% of the excitatory synaptic boutons in late adolescent ferret contain spinules.

## Materials and Methods

### Animals

A total of ten male and female ferrets were used for this study, postnatal day (p)21 (*n* = 1), p28 (*n* = 1), p46 (*n* = 1), p47 (*n* = 1), p60 (*n* = 2), p66 (*n* = 1), >p90 (*n* = 3). These ages were chosen because they are key developmental time points: before eye-opening (i.e., before ∼p32), the peak of the critical period for ocular dominance and TC axon morphological plasticity (∼p46), the end of this critical period in ferret binocular V1 (∼p60), and ages approaching ferret sexual maturity (>p90; Issa et al., 1999). For the purposes of 2D analyses, measurements from animals at similar developmental time points were grouped [i.e., p21–p28 (*n* = 2), p46–p47 (*n* = 2), p60–p66 (*n* = 3), and >p90 (*n* = 3)], after determining that intragroup (e.g., p21 vs p28) measurements of synapse length and bouton area were not statistically significant (one-way ANOVA and *post hoc* Bonferroni corrected *t* tests, *p* > 0.1; data not shown). All animal procedures and protocols were in accordance with NIH guidelines for humane handling of animals and were approved by the Institutional Animal Care and Use Committee at the University of Virginia.

### Perfusions

Animals were given an overdose of Nembutal (in excess of 50 mg/kg) and were perfused transcardially with 4% paraformaldehyde and 0.5% glutaraldehyde in 0.1 m phosphate buffer (PB; pH 7.4). To prevent the reported increase in spinule prevalence because of longer perfusion times (Tao-Cheng et al., 2009), we only included animals in this study where perfusion time from thorax incision to fixation was <100 s. Following perfusions, brains were removed and kept in 4% paraformaldehyde overnight at 4°C. On the following day, each brain was dissected, occipital lobe blocks were placed in a vibratome, and coronal sections of V1 were cut at 60 μm. Floating coronal sections were immediately treated with 1% sodium borohydride, rinsed five to six times in PBS, and free-floating sections were stored in PBS containing 0.05% sodium azide at 4°C.

### Transmission electron microscopy (TEM)

Sections prepared for TEM examination were rinsed in 0.1 m PB and then immersed in 1% osmium tetroxide (in 0.1 m PB) for 1 h. Next, sections were rinsed in 0.1 m PB, dehydrated in a series of ethanol dilutions, and then incubated in 4% uranyl acetate (in 70% ethanol) at 4°C overnight. Sections were then dehydrated in acetone and incubated in a series of three progressively concentrated acetone-Epon 812 resin (EMS, catalog #RT14120) mixtures, each for 4 h – overnight at room temperature. Next, sections were flat embedded between two pieces of plastic film (EMS, catalog #50425-10) and placed in a 60°C oven overnight. Areas within layer four of the binocular region of V1 of embedded coronal sections were marked under light microscopy using established white matter landmarks (Law et al., 1988), excised from the flat embed, placed in a capsule (EMS, catalog #70010-B), and filled with Epon 812 resin. Layer four of binocular V1 is a site of robust physiological and morphological developmental plasticity (Issa et al., 1999; Coleman et al., 2010; Maffei et al., 2010). Next, tissue blocks were cured in a 60°C oven for 36–48 h or until polymerized. Outlines and landmarks of layer four of binocular V1 were then drawn using a camera lucida and this area of interest was cut into a trapezoid on the EPON block. Ultrathin 60 to 70 nm-thick sections were then cut using an ultramicrotome (Leica Ultracut, Leica Microsystems) and collected on TEM copper and nickel mesh grids. Ultrathin sections were viewed using a Philips CM 100 or JEOL JEM 1400 TEM equipped with a 2k x 3k Olympus Morada and a 2k x 2k Gatan Ultrascan 1000XP camera, respectively. Digital electron micrographs were taken at final magnifications on these TEMs to achieve ∼0.5 nm/pixel resolution. Potential tissue shrinkage because of TEM/focused ion beam scanning electron microscopy (FIBSEM) tissue processing was not expected to differentially affect asymmetric synapses across the age groups examined (Korogod et al., 2015) and was not corrected for in our analyses.

Ultrathin sections were selected at random from each TEM grid and nonoverlapping TEM images were taken across L4 within each ultrathin section. To ensure that we did not image the same synapse more than once, imaged ultrathin sections at each age were taken ≥2 μm apart in depth (z), which was ∼3.5 times greater than the depth of the largest postsynaptic density (PSD) from our FIBSEM analyses. In each ultrathin section, we analyzed every Gray’s Type I “asymmetric” excitatory synapse (Gray, 1959) we encountered, being careful to locate and include smaller synapses within these sections in our analyses. Putative excitatory synapses were only included in our analyses if they (1) had parallel alignment of presynaptic and postsynaptic membranes at the active zone, (2) had more than or equal to three presynaptic vesicles, and (3) had a prominent asymmetric PSD opposite these presynaptic vesicles. All excitatory synapses meeting these criteria were analyzed using FIJI (Schindelin et al., 2012) software to record their bouton areas, synapse lengths, and spinule profile areas (if present).

In 2D TEM images, spinules were identified as double membrane-bound structures (i.e., inner spinule lipid bilayer and outer bouton lipid bilayer) within excitatory synaptic boutons. In addition, spinules were only included in our analyses if they were ≥2.5 times the size (diameter of shortest spinule axis) of the averaged sized synaptic vesicle at that age and if their interiors were electron translucent. We adopted these conservative criteria to differentiate spinules from synaptic vesicles, elongated endoplasmic reticula (Wu et al., 2017), and relatively electron opaque lysosomes (Marty and Peschanski, 1994) in our 2D TEM images. In the event that multiple spinule 2D profiles were present within a single SBB, these spinule areas were summed for the purposes of our spinule area analyses.

### FIBSEM

Tissue blocks from a p21, p46, p60, and >p90 animal, originally used in our 2D TEM analyses, were retrimmed and prepared for FIBSEM. First, a series of 60 nm ultrathin sections were cut to a depth of ∼3 μm from each block to ensure we would be analyzing novel synapses in our 3D analyses. All processing and imaging of FIBSEM blocks was performed at the Multiscale Microscopy Core at Oregon Health and Sciences University. Tissue blocks were mounted onto standard 12.5 mm flat SEM stubs (Ted Pella, catalog #16111) using Leitsilber 200 silver paint (Ted Pella, catalog #16035). The resulting blocks were then trimmed using a Trim90 diamond knife (Diatome), and the final sample blocks were coated with 8 nm of carbon using a Leica ACE 600 unit. Regions of interest from the block faces were imaged using Thermo Fisher Scientific’s Auto Slice & View software package to automate the serial sectioning and data collection processes. Each block face was scanned on a FEI Helios 660 DualBeam SEM at a 52° tilt and with a 4.2 mm working distance. For each slice, 4 nm of resin was removed at 30 keV and 0.79 nA. Each slice was imaged with a 3-kV acceleration voltage and 400-pA current in backscatter mode with inverse contrast using the in-column detector. Images of 6144 × 4096 pixels were acquired at a resolution of 4 nm/pixel.

We obtained aligned 4 nm isotropic FIBSEM image stacks from a p21, p46, p60, and >p90 animal. Image stacks of L4 V1 were of the following volumes: p21 (15.1 × 14.1 × 2.8 μm), p46 (9.7 × 8.4 × 2.7 μm), p60 (24.2 × 16.2 × 2.4 μm), and >p90 (24.2 × 16.2 × 2.4 μm). All serial image stacks were analyzed in FIJI. We located every excitatory synapse meeting our excitatory synapse criteria within each image stack and analyzed them in 3D for (1) the presence/absence of a spinule, (2) presence/absence of a perforated PSD, and (3) the origin of the spinule (if present). Importantly, spinules were only included in our FIBSEM analyses if they could be observed invaginating into a presynaptic bouton and were then observed as membrane-bound structures (i.e., unambiguous visualization of lipid bilayer of spinule surrounded by bouton’s lipid bilayer) within the presynaptic bouton in at least one image. This additional positive spinule identification criteria necessarily differs from our 2D analysis criteria, since 4 nm FIBSEM z-resolution allows one to visualize a spinule’s invagination site into each SBB. In contrast, our 2D analyses required stricter size criteria to identify spinules from 2D profiles (i.e., ≥2.5 times a synaptic vesicle), because of a lack of information on the potential origins of membrane-bound objects within 2D TEM images. Therefore, our 2D analyses excluded some number of smaller spinules and consequently yielded reduced spinule prevalence. Spinule origins in FIBSEM stacks were determined by tracking them within the image volume to their parent neurite/glia. When it was not possible to identify the parent neurite/glia from which a spinule emerged (e.g., because of section loss/defect, or an object lacking one or more identifiable morphological criteria by the end of the image volume) we assigned this spinule to the “unknown” origin category. Following quantification of spinule origins, we reconstructed SBBs from representative parent origins, including their synaptic partners and spinule parent neurites/glia, using Reconstruct software (Fiala, 2005). 3D reconstruction surfaces were then remeshed and rendered semi-transparent for spinule and PSD visualization using Blender open-source software. PSDs were deemed “perforated” if they displayed one or more separations in the electron-dense region of the PSD, whereas macular PSDs were defined as PSDs bereft of these separations. PSD separations needed to be seen in the same *x*/*y* location in at least two consecutive serial FIBSEM images (i.e. 8 nm in the *z* dimension) to count as perforations.

### Statistics

Descriptive statistics for all group comparisons in this study are listed in Tables 1-3. For 2D TEM intragroup comparisons of spinule area, bouton area, and synapse length within developmental age groups, we performed one-way ANOVAs (p60–p p66 and >p90 age groups) and *post hoc* Bonferroni corrected *t* tests (p21–p28 and p46–p47 age groups) to compare means from animals within each group and found that there were no significant differences (*p* > 0.1) between animals within any single age group (data not shown). Accordingly, we grouped 2D data within each age group into a single group mean. To compare spinule area, bouton area, and synapse length between developmental groups, we then performed one-way ANOVAs, followed by Bonferroni *post hoc* two-tailed *t* tests if our ANOVA results were significant at the *p* < 0.05 level (Bonferroni correction set at *p* < 0.0168). To compare spinule prevalence within 2D TEM images between age groups, we used two-tailed χ^2^ tests with Bonferroni correction (4 × 2 and 2 × 2 contingency tables, three comparisons, Bonferroni corrected significance level set at *p* < 0.0168). For our Gardner–Altman estimation statistic plots (Gardner and Altman, 1986), morphological measurement distributions were compared using non-parametric Mann–Whitney *U* tests.

For FIBSEM analyses, to compare the proportions of SBBs, perforated PSDs, and spinule origins between animals, we used two-tailed χ^2^ tests with Bonferroni correction (4 × 2 and 2 × 2 contingency tables, three comparisons, Bonferroni corrected significance level set at *p* < 0.0168). For all figures, ***p < 0.001, **p < 0.015, *p < 0.05.

## Results

To determine whether the onset of sensory activity or cortical plasticity state correlate with SBB prevalence (i.e., the number of SBBs divided by the total number of sampled excitatory presynaptic boutons), we analyzed excitatory synaptic boutons within L4 from ferret V1 across key developmental stages. We reasoned that if the onset of sensory activity and/or heightened cortical plasticity is primarily responsible for increasing the proportion of SBBs in V1, SBB prevalence should increase rapidly between when V1 cells first receive sensory activity (i.e., eye-opening) and when Hebbian plasticity is maximally expressed in V1 (∼p32–p46 in ferret; Issa et al., 1999). In contrast, if the presence of a spinule within a presynaptic bouton is related to its physiological maturation and morphological stability (Cheetham and Fox, 2010; Smith et al., 2015; Qiao et al., 2016), we expect to see an increase in SBB prevalence in parallel with this progressive functional maturation. To this end, we prepared sections from L4 of binocular V1 for TEM from ferrets at key postnatal ages: p21–p28 (before eye-opening; *n* = 2 animals), p46–p47 (height of plasticity in V1; *n* = 2 animals), p60–p66 (end of critical period in V1; *n* = 3 animals), and >p90 (ferrets between p90 and p150 that were nearing sexual maturity; *n* = 3 animals). We took non-overlapping 2D TEM images within L4 from animals within each of these age groups and quantified morphological features correlated with synaptic strength (Murthy et al., 2001; Ostroff et al., 2002; Branco et al., 2010; Holderith et al., 2012; Araya et al., 2014; Meyer et al., 2014), in addition to documenting the presence or absence of SBBs, at every excitatory synapse we encountered (see Materials and Methods).

### Morphometry of excitatory synaptic boutons and spinules across development

**Figure 1.**
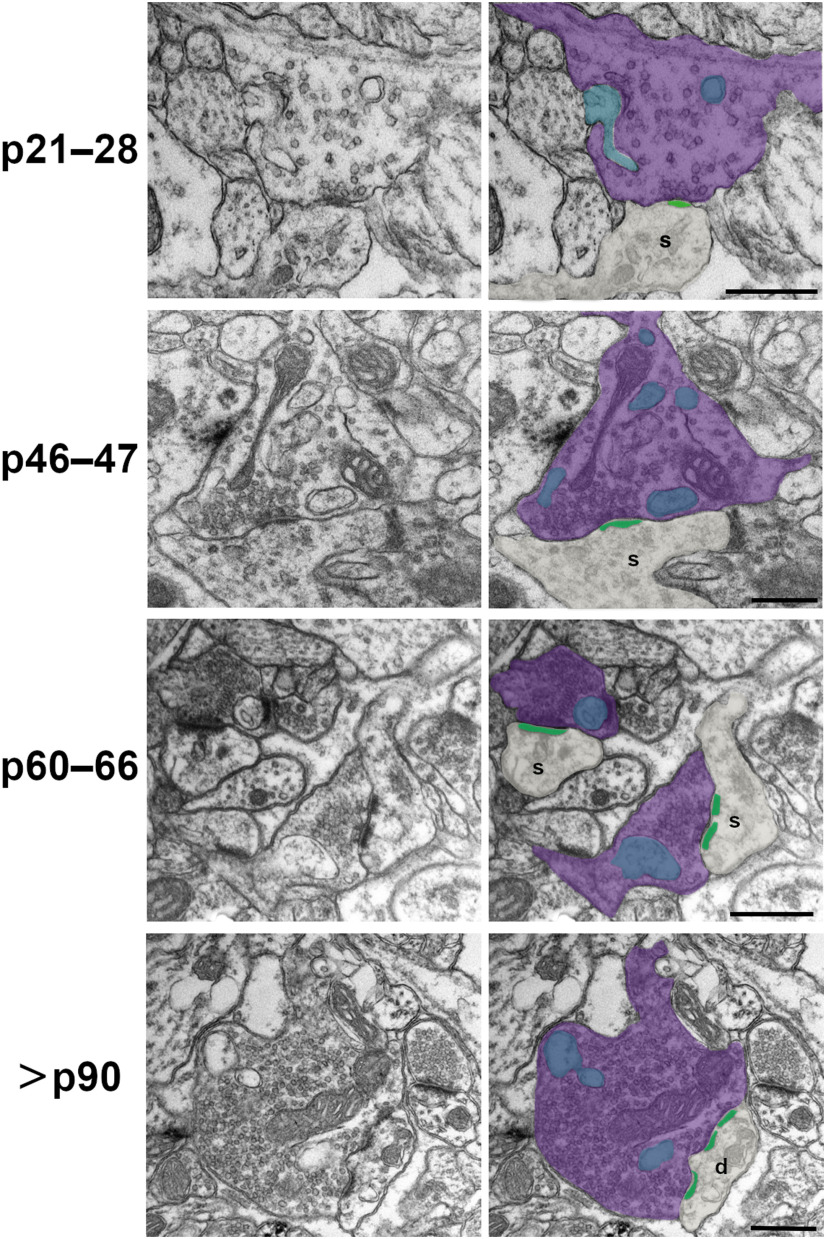
Development of excitatory SBB morphology. Spinules (blue) were observed within cortical boutons (purple) at every postnatal day age group examined. In 2D TEM sections, spinules were occasionally observed invaginating into SBB profiles (e.g., p21–p28); however, most spinules appeared as ovoid or circular double membrane-bound structures encapsulated within their “host” bouton. PSDs (green) at SBB synapses sometimes contained perforations (e.g., p60–p66 and >p90 panels). Note that SBBs at times displayed multiple spinule cross-sections (i.e., 2D profiles of putative spinules), yet in our 2D TEM analyses, it was not possible to attribute these to single spinules with complex morphologies, or to multiple spinules protruding into a single SBB. s = postsynaptic spine; d = postsynaptic dendrite. Scale bars = 0.5 μm.

**Figure 2.**
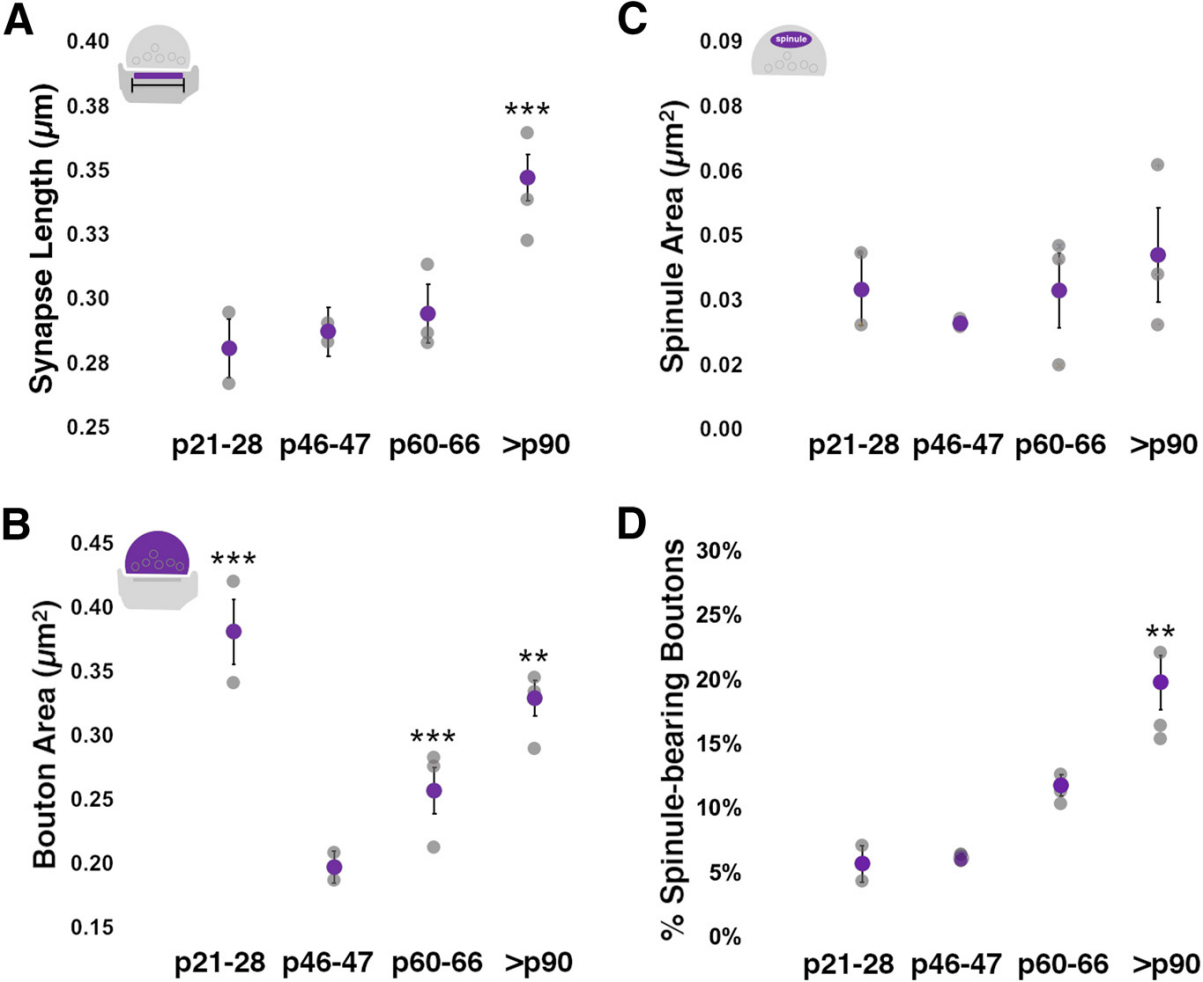
Excitatory bouton areas and spinule prevalence increase over development. ***A***, 2D synapse profile length, as defined by the PSD, remains relatively stable from before eye-opening (p28) until the end of the critical period for plasticity in ferret visual cortex (p60). However, PSD length increases substantially from the end of the critical period until at least the cusp of maturity (>p90 ages). ***B***, Excitatory presynaptic bouton areas are largest at a time when most boutons appear to have elongated en passant morphology (p21–p28). Bouton size decreases significantly by p46–p47 and then shows a steady increase until at least p90. ***C***, Average spinule profile areas do not appreciable change across the postnatal ages examined. Note that spinule areas show relatively large interanimal variation attributed to the variation in spinule sizes from distinct parent neurite/glia origins. ***D***, Excitatory SBBs are most prevalent in late adolescence (>p90) versus at the height (p46–p47) or end (p46–p47) of the critical period for plasticity in ferret V1. A trend toward increased SBB prevalence is also seen between p46–p47 and p60 ages. For ***A–D***, statistical comparisons were performed between p21–p28 and p46–p47, p46–p47, and p60–p66, and p60–p66, and >p90 (for details, see Table 3). Gray circles = individual animal means; Purple circles = group means ± SEM; **p < 0.015, ***p < 0.001.

Qualitatively, p21–p28 excitatory synapses had less pronounced PSDs and often displayed en passant bouton morphology (Fig. 1), whereas at older ages PSDs appeared to become larger and thicker and excitatory boutons displayed more varied and complex morphologies. Indeed, while our quantitative analyses revealed that 2D synapse lengths did not appreciably change from p21 to p66, PSD lengths did show a significant 16% increase between p60–p66 and >p90 ages (Fig. 2*A*; Tables 1, 3). Cortical synaptic boutons have been shown to increase in size over postnatal development (Erisir and Dreusicke, 2005). Yet interestingly, we found that 2D bouton areas were largest at p21–p28 (before eye-opening), dropped by 93% at p46–p47, and then increased significantly until >p90 (Fig. 2*B*). This initial developmental decrease in bouton areas has been reported previously for excitatory synaptic boutons in L4 (Dufour et al., 2016), and may stem from a preponderance of elongated en passant boutons at early developmental ages. Following this initial decrease however, excitatory boutons in L4 showed a steady, nearly linear increase in their 2D profile areas from p46 to >p90 (Fig. 2*B*; Extended Data Fig. 2–1; Tables 1, 3).

**Table 1:** Means, SE, and sample sizes for 2D TEM morphological analyses

	Age	Total boutons	Total synapses	PSD length (μm)	Bouton area (μm^2^)	% SBBs	Spinule area (μm^2^)	Spinule:bouton area (%)

	p21	70	74	0.29	0.34	4%	0.04	12%
	p28	71	78	0.27	0.42	7%	0.02	6%
Group sum Group mean ± SEM (*n* = animals)		**141**	**152**	0.28 ± 0.01	0.38 ± 0.03	6 ± 1.4%	0.03 ± 0.01	9 ± 3.1%
Cumulative mean ± SEM (*n* = sampled structures)				**0.28 ± 0.01**	**0.38 ± 0.03**	**6 ± 1.4%**	**0.03 ± 0.01**	**6 ± 1.5%**
								
	p46	102	102	0.28	0.19	6%	0.02	6%
	p47	94	94	0.29	0.21	6%	0.03	12%
Group sum Group mean ± SEM (*n* = animals)		**196**	**196**	0.29 ± 0.01	0.20 ± 0.01	6 ± 0.3%	0.03 ± 0.001	9 ± 3.2%
Cumulative mean ± SEM (*n* = sampled structures)				**0.29 ± 0.01**	**0.20 ± 0.01**	**6 ± 0.3%**	**0.02 ± 0.004**	**8 ± 3.2%**
								
	p60_1	25	29	0.28	0.21	10%	0.04	14%
	p60_2	111	113	0.29	0.28	13%	0.01	4%
	p66_1	118	124	0.31	0.28	11%	0.04	14%
Group sum Group mean ± SEM (*n* = animals)		**254**	**266**	0.30 ± 0.01	0.26 ± 0.02	11 ± 0.8%	0.03 ± 0.01	11 ± 3.4%
Cumulative mean ± SEM (*n* = sampled structures)				**0.30 ± 0.01**	**0.27 ± 0.01**	**11 ± 0.8%**	**0.03 ± 0.01**	**10 ± 1.7**
								
	>p90_1	61	61	0.32	0.29	16%	0.06	16%
	>p90_2	149	150	0.36	0.33	15%	0.02	5%
	>p90_3	109	112	0.34	0.35	22%	0.04	4%
Group sum Group mean ± SEM (*n* = animals)		**319**	**323**	0.35 ± 0.01	0.32 ± 0.01	18 ± 2.1%	0.04 ± 0.01	8 ± 4%
Cumulative mean ± SEM (*n* = sampled structures)				**0.35 ± 0.01**	**0.33 ± 0.01**	**18 ± 2.1%**	**0.04 ± 0.01**	**8 ± 1%**

Means and SEM for individual animals and developmental age groups are listed for each 2D TEM analysis measurement (column titles). Population sizes are listed for individual animals and age groups under total boutons and total synapses columns. % SBBs = percentage of sampled excitatory presynaptic boutons that contained spinules. Spinule: bouton area % = percentage of bouton area occupied by spinule area.

10.1523/ENEURO.0426-19.2020.f2-1 Extended Data Figure 2-1 Estimation statistics plots showing mean differences between developmental synapse length and bouton area comparisons. ***A***, Gardner–Altman estimation plot (Gardner and Altman, 1986) showing the raw synapse profile length values for >p90 and p60–p66 groups (left *y*-axis), as well as the mean difference (black dot near right *y*-axis), and the bootstrap 95% confidence interval (vertical error bar from black dot). Mann–Whitney *U* test, *p* = 1.5 × 10^−4^. ***B***, Estimation plot showing raw excitatory bouton areas for all developmental age groups examined. Plots marked as in ***A***, except that mean differences between groups and confidence intervals are shown below each comparison. Mann–Whitney *U* test, *p* = 0.004 (p60–p66 vs >p90), and 2.8 × 10^−14^ (p21–p28 vs p46–p47). Download Figure 2-1, TIF file.

In 2D TEM images, spinule cross-sections (i.e., 2D sections of embedded putative spinules) were present within excitatory synaptic boutons at every age examined. Spinule cross-sections within excitatory boutons displayed two lipid bilayers (outer bouton and inner spinule membranes) and were distinguished from other cellular organelles (see Materials and Methods). In measuring spinule areas within SBBs across postnatal development, we found that average spinule area remained relatively constant but showed a trend that mirrored the development of bouton areas from before eye-opening until after p90 (Fig. 2*C*; Tables 1, 3). Moreover, on average spinules occupied ∼6–11% of their encapsulating bouton’s area over development, and this relationship held steady until after p90 (Table 1). Yet despite this relatively stable spinule area to bouton area ratio, the SBB population at >p90 had larger bouton areas than >p90 boutons without spinules (Extended Data Fig. 2–2; Table 3). These data suggest that spinule engulfment increases SBB size over cortical boutons without spinules and that a mechanism may exist to constrain spinule size as they invaginate into larger SBBs over development.

10.1523/ENEURO.0426-19.2020.f2-2 Extended Data Figure 2-2 SBBs are larger than boutons without spinules (no spinule). ***A***, Histogram showing the distribution for the percentage of >p90 SBBs (purple) and >p90 presynaptic boutons without spinules (blue) with various sized areas. Inset, Cumulative histogram of this same comparison. ***B***, Estimation plot showing the raw data, mean difference (black dot), and bootstrapped 95% confidence interval (lines extending from dot) for areas from >p90 SBBs and boutons without spinules. Mann–Whitney *U* test, p = 2.7 × 10^−9^; ***p < 0.001. Download Figure 2-2, TIF file.

### Spinule prevalence across development: 2D TEM analyses

We hypothesized that if spinule engulfment by SBBs were driven by the onset of sensory activity or a heightened state of cortical plasticity, SBBs should increase in prevalence between eye-opening (∼p30) and the height of Hebbian and homeostatic plasticity in V1 (∼p46–p47; Issa et al., 1999; Yu et al., 2011). Furthermore, we predicted that if spinule engulfment by cortical boutons were correlated with the state of V1 plasticity, SBBs should be most prevalent around p46–p47 and then should slowly wane in parallel with the expression of plasticity in ferret binocular V1 (Issa et al., 1999; Li et al., 2006). In contrast, if spinules play a role in stabilizing functionally and morphologically mature synapses, SBB prevalence should continue to increase as the expression of cortical plasticity wanes. We analyzed 910 excitatory presynaptic boutons across ferret postnatal developmental for the presence of spinules and found that the percentage of SBBs in L4 did not appreciably change from before the onset of visual experience until the canonical peak of plasticity in V1 (Fig. 2*D*; Tables 1, 3).

In contrast, the percentage of SBBs trended toward increased levels between the height (∼p46–p47) and end of the canonical critical period in V1 (p60–p66; *p* = 0.041, not significant), and the proportion of cortical boutons containing a spinule significantly increased between p60–p66 and >p90 (68% increase; *p* = 0.009; Fig. 2*D*; Tables 1, 3). Thus, although deprivation-induced cortical plasticity within ferret V1 decreases by ∼50% between p42 and p60, and deprivations after p100 fail to induce measurable ocular dominance plasticity (Issa et al., 1999), the percentage of SBBs within ferret V1 increase significantly over this same time frame. Taken together, these data suggest that spinule encapsulation by developing cortical SBBs is regulated by processes that parallel the physiological and morphological maturation and presynaptic stability of V1 synapses (Jones and Calverley, 1991; Durack and Katz, 1996; Erisir and Dreusicke, 2005; Cheetham and Fox, 2010; Smith et al., 2015; Qiao et al., 2016) and that SBB prevalence is not correlated with the onset of sensory activity or with the morphological malleability permitted during heightened states of neocortical plasticity.

### 3D FIBSEM analyses of SBBs in V1

**Figure 3.**
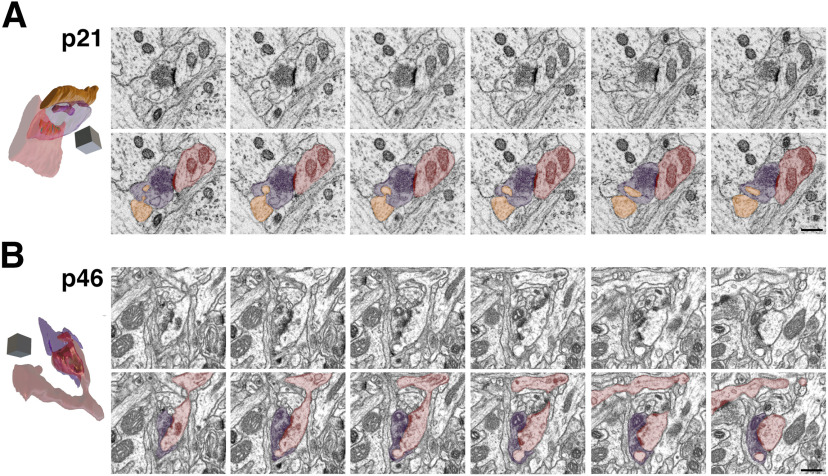
FIBSEM images of SBBs in p21 and p46 V1. ***A***, Adjacent FIBSEM images of an SBB in L4 of V1 from a p21 ferret. FIBSEM images are ∼25 nm apart in z (depth) on average, picked to display the progression of spinule invagination and engulfment by the SBB. Top panel, Raw FIBSEM images. Bottom panel, Pseudo-colored to highlight SBB (purple), adjacent axon projecting a spinule (orange), and postsynaptic dendrite (red). Left, Full reconstruction of this SBB showing transparent bouton with engulfed adjacent axon spinule and postsynaptic dendrite. Note the macular shaped PSD (green) formed between the SBB and the red dendrite. Identical SBB as shown in Figure 5*A*,*A1*. ***B***, Adjacent FIBSEM images of an SBB in L4 of V1 from a p46 ferret. Top and bottom panels arranged and colored as in ***A***, showing a postsynaptic spine (red) projecting a spinule into its presynaptic SBB partner (purple). Left, Full reconstruction of this p46 SBB showing postsynaptic spine spinule enveloped by its presynaptic bouton. Note the perforated complex shaped PSD (green). Identical SBB as shown in Figure 5*D*,*D1*. Scale bars = 0.5 μm; 3D scale cubes = 0.5 μm^3^.

**Figure 4.**
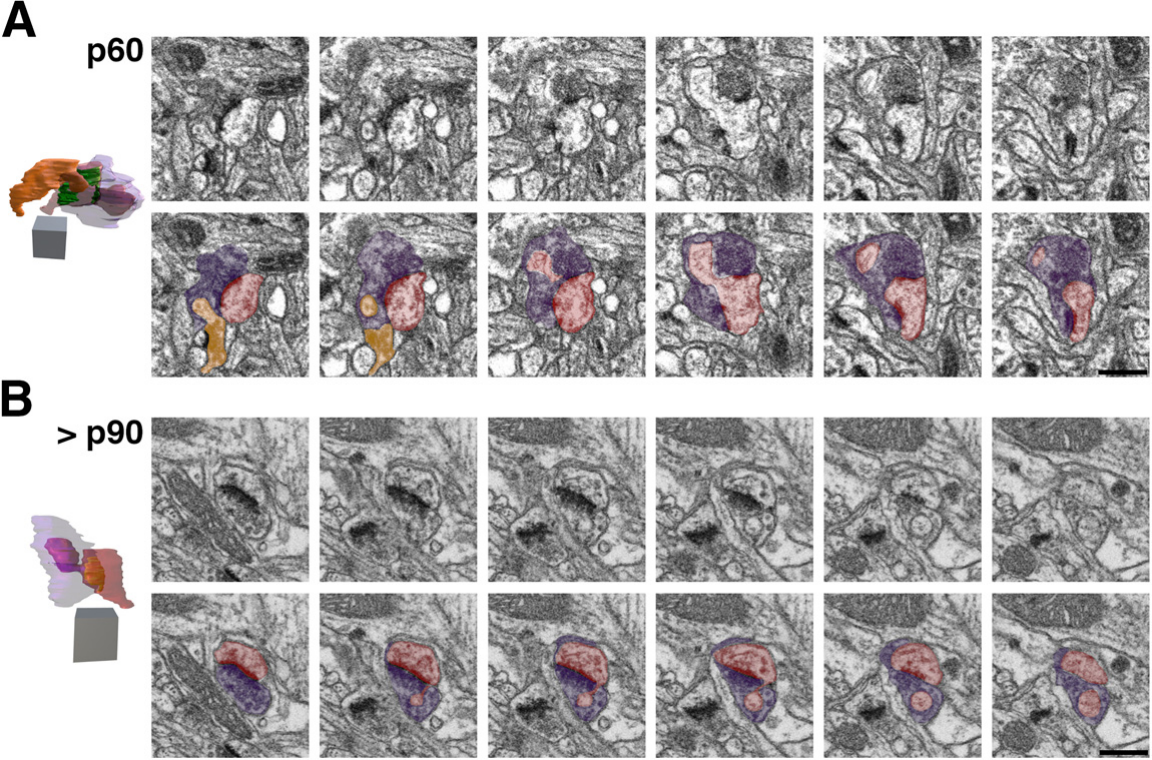
FIBSEM images of SBBs in p60 and >p90 V1. ***A***, Adjacent FIBSEM images of an SBB in L4 of V1 from a p60 ferret. FIBSEM images are ∼25 nm apart in z (depth) on average, picked to display the progression of spinule invagination and engulfment into the SBB. Top panel, Raw FIBSEM images. Bottom panel, Pseudo-colored to highlight SBB (purple), adjacent axon/bouton (orange), and postsynaptic spine (red). Note the adjacent bouton (orange) with a synapse onto a spine that protrudes a spinule into this SBB at the bottom left of the first image in the series, and the spinule from the postsynaptic spine that invaginates into this SBB across the middle of its perforated PSD. Left, Full reconstruction of this SBB showing transparent bouton (purple) with engulfed postsynaptic spine (red) and adjacent bouton (orange) spinules. Note the horseshoe-shaped perforated PSD. Identical SBB as shown in Figure 6*C*,*C1*. ***B***, Adjacent FIBSEM images of an SBB in L4 of V1 from a >p90 ferret. Top and bottom panels arranged and colored as in ***A***. Note the postsynaptic spine (red) that sends its spinule into its SBB (purple) partner from the edge of the PSD. Left, Full reconstruction of this SBB (purple), made transparent to show the engulfed anchor-like spinule from its postsynaptic spine partner. Macular-shaped PSD (green) appears yellow within spine. Identical SBB as shown in Figure 7*D*,*D1*. 2D scale bars = 0.5 μm; 3D scale cubes = 0.5 μm^3^.

**Figure 5.**
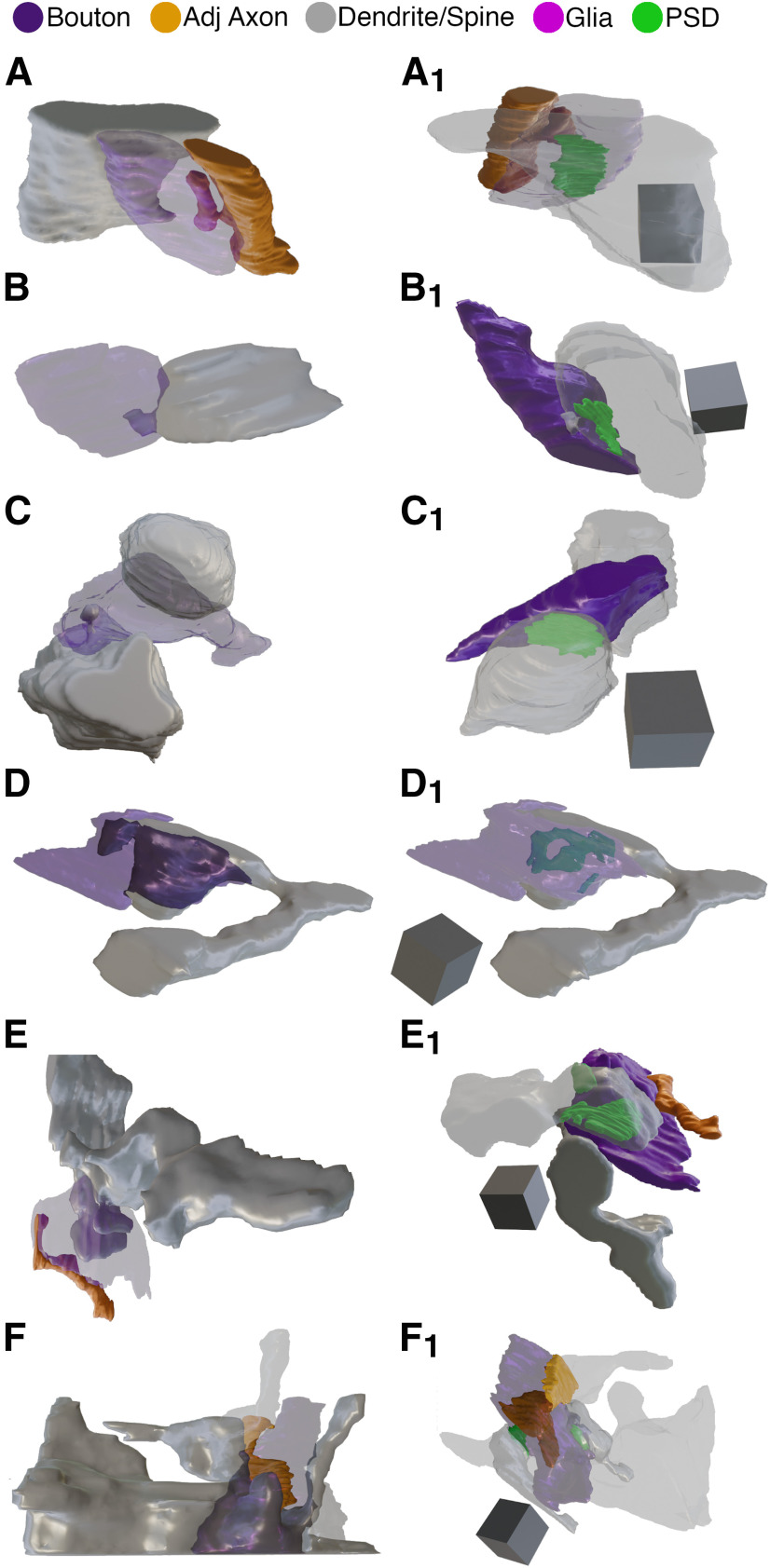
3D Reconstructions of p21 and p46 SBBs. Focused ion beam serial electron microscopy 3D reconstructions of SBBs (purple) and their spinules from axon/boutons (yellow), and spines/dendrites (gray). Object color scheme shown at top. ***A–C***, 3D reconstructions from a p21 ferret showing spinules from an adjacent axon (***A***, ***A_1_***), postsynaptic dendrite (***B***, ***B_1_***), and adjacent dendrite (***C***, ***C_1_***) projecting into L4 SBBs. Note that B_1_ shows the spinule emerging from the edge (below and left) of the PSD. Reconstruction shown in A is the identical SBB shown in Figure 3*A*. ***D–F***, 3D reconstructions from a p46 ferret showing hook-like spinule from a postsynaptic spine (***D***, ***D_1_***), and spinules from adjacent axons and adjacent dendrites (***E***, ***E_1_***, ***F***, ***F_1_***) projecting into L4 SBBs. Note the complex perforated PSD in ***D1***, the identical SBB shown in Figure 3*B*. In ***F***, ***F_1_***, an SBB with synapses (green) onto two postsynaptic spines receives a large adjacent dendrite spinule (***F***) and a large adjacent axon spinule (***F_1_***). ***A_1_–F_1_***, Identical SBBs as shown to the left in ***A–F***, but with transparent postsynaptic neurites and/or SBBs to highlight the morphology and locations of the PSDs (green) or spinules. 3D scale cubes = 0.5 μm^3^.

**Figure 6.**
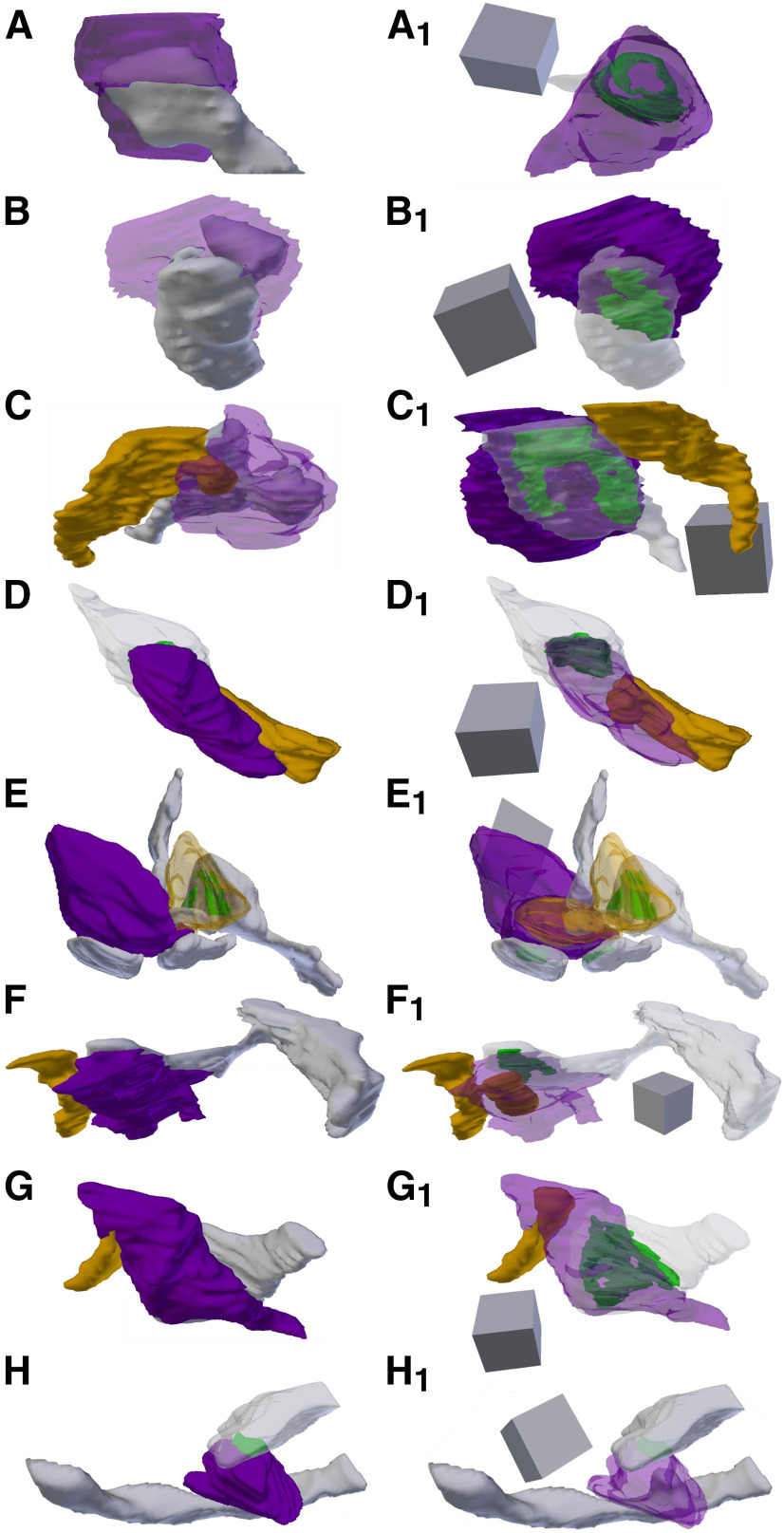
3D Reconstructions of p60 SBBs. Focused ion beam serial electron microscopy 3D reconstructions of SBBs (purple) and their spinules from axon/boutons (yellow), and spines/dendrites (gray). Object color scheme as shown in Figure 5. ***A–C***, Reconstructions of a postsynaptic spine head (***A***) and postsynaptic spine anchor-like spinules (***B***, ***C***) projecting into L4 p60 SBBs. Note that in ***C***, a second spinule from a synaptic bouton (orange) projects into the “upper” portion of the SBB (same bouton shown in Fig. 4*A*). ***C–G***, Reconstructions showing axons/bouton spinules of various sizes engulfed by p60 SBBs. Note that in ***E***, a presynaptic bouton (orange) receives a spinule from its postsynaptic spine partner and a portion of this bouton with its engulfed spinule protrude into a large adjacent SBB (purple) with synapses (green) onto three postsynaptic spines. ***H***, An SBB with a synapse onto a postsynaptic dendrite (top) receives a spinule from an adjacent dendrite (bottom). ***A_1_–H_1_***, Identical SBBs as shown to the left in ***A–H***, but with transparent postsynaptic neurites and/or SBBs to highlight the morphology and locations of the PSDs (green) or spinules. 3D scale cubes = 0.5 μm^3^.

**Figure 7.**
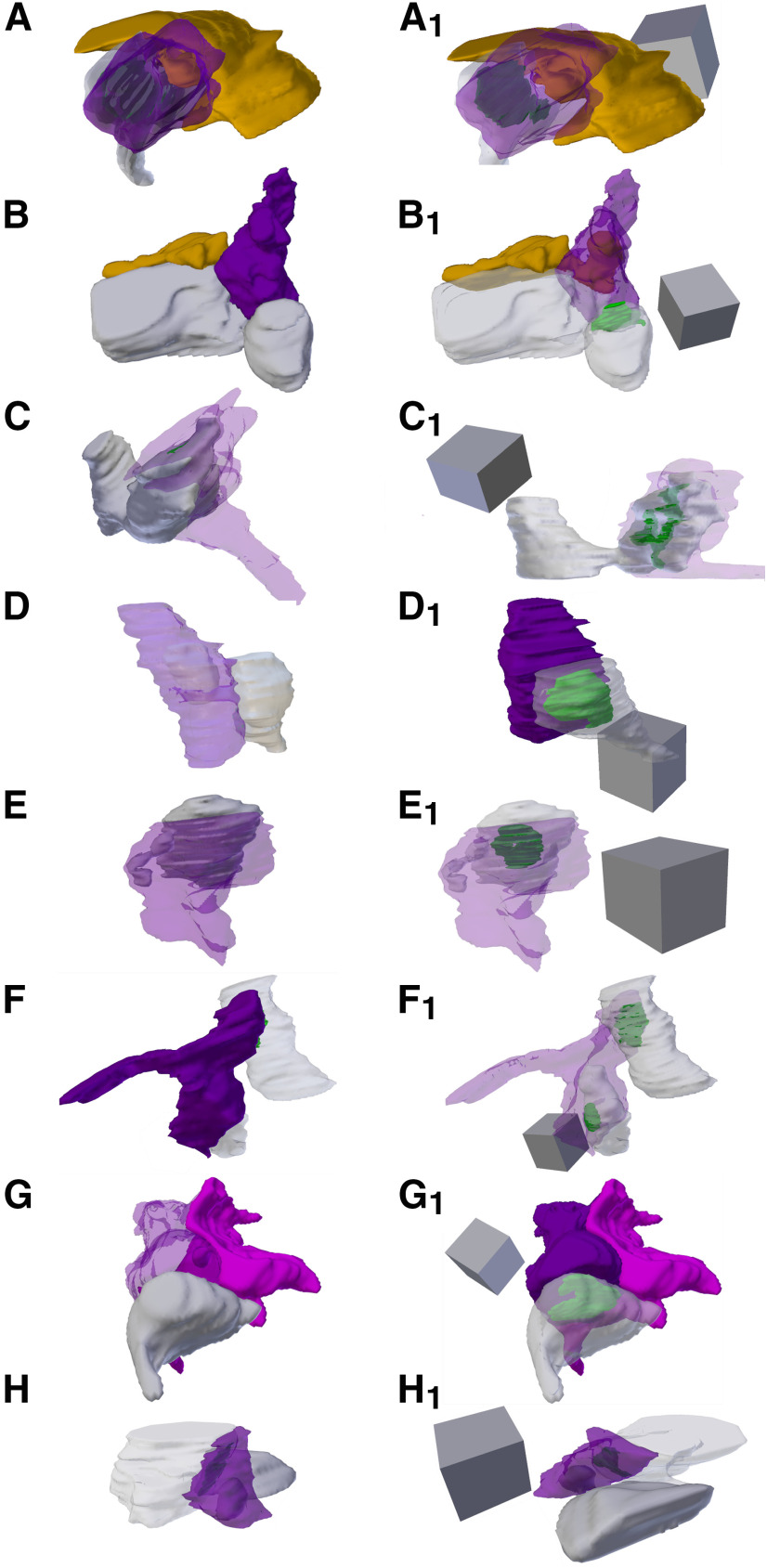
3D Reconstructions of >p90 SBBs. Focused ion beam serial electron microscopy 3D reconstructions of SBBs (purple) and their spinules from axon/boutons (yellow), spines/dendrites (gray), and glia (pink) from L4 of V1 of a >p90 ferret. Object color scheme as shown in Figure 5. ***A***, ***B***, Reconstructions showing adjacent axons/bouton spinules engulfed by SBBs with synapses onto postsynaptic spines. ***C–F***, Reconstructions of postsynaptic spine heads (***C***, ***F***), and anchor-like spinules (***D***, ***E***) projecting into their SBB partners. Note the horseshoe-shaped perforated PSD (green) in ***C_1_***, and the SBB shown in ***F***, ***F_1_*** enveloping approximately two-thirds of its postsynaptic spine partner. Reconstruction shown in ***D*** is the identical SBB shown in Figure 4*B*. ***G***, An SBB with a synapse onto a postsynaptic spine receives a spinule from an adjacent glia. ***H***, An SBB with a synapse onto a postsynaptic dendrite engulfs a spinule from an adjacent dendrite. ***A_1_–H_1_***, Identical SBBs as shown to the left in ***A–H***, but with transparent postsynaptic neurites and/or SBBs to highlight the morphology and locations of the PSDs (green) or spinules. 3D scale cubes = 0.5 μm^3^.

We took advantage of both 2D TEM and 3D FIBSEM in a complementary approach to determine the time course for the developmental expression of SBBs in L4 of V1. Our 2D TEM analyses were designed to quantify a large number of excitatory boutons across multiple animals and ultrathin sections per age group to capture the potential variability in SBB prevalence and quantitative morphometry across individual animals and across the depth of L4. However, while they have higher lateral resolution, 2D TEM analyses are biased toward omitting smaller objects (i.e., smaller boutons and spinules; Colonnier and Beaulieu, 1985). Moreover, because of a larger physical section thickness (i.e., ∼50–80 vs 4 nm in FIBSEM), 2D TEM analyses are not suitable for quantifying small (e.g., 10–30 nm) perforations in PSDs, and analyses of single TEM images cannot be used to determine the neurite/glial source of spinules embedded within SBBs. Thus, to determine the origin of spinules within SBBs, the potential relationship between an invaginating spinule and perforations in PSDs, and ascertain the accuracy of our 2D analyses of SBB prevalence, we prepared one tissue block from each developmental age group (i.e., p21, p46, p60, and >p90) for FIBSEM. We obtained 4-nm/pixel isotropic FIBSEM image stacks from L4 of V1 from each animal, yielding aligned image volumes from p21 (15.1 × 14.1 × 2.8 μm), p46 (9.7 × 8.4 × 2.7 μm), p60 (24.2 × 16.2 × 2.4 μm), and >p90 (24.2 × 16.2 × 2.4 μm) brains. We evaluated every excitatory synapse we encountered (Table 3) within each image volume in 3D for the presence/absence of a spinule within synaptic boutons, the origin of the invaginating spinule (if present), and the presence/absence of a perforated PSD. In addition, we reconstructed a total of 22 SBBs along with their invaginating spinules, postsynaptic targets, and PSDs (Figs. 3-7). With 4-nm isotropic pixel resolution we were able to follow every spinule back to its parent neurite, as well as determine the percentages of PSDs that contained perforations. Since perforations in the PSD of cortical synapses have been found to correlate with measures of synaptic plasticity (Calverley and Jones, 1990; Geinisman et al., 1993; Toni et al., 1999; Ganeshina et al., 2004), and potentially with the prevalence of SBBs (Jones et al., 1991; Spacek and Harris, 2004), we evaluated each synapse for the presence of perforated PSDs.

We encountered a range of neurites that projected spinules into L4 SBBs, including postsynaptic spines, adjacent (i.e., not synaptic with the SBB) spines, adjacent dendrites, adjacent axons/boutons, and glial processes, in line with previous reports on the variety of spinule-projecting objects in hippocampus (Sorra et al., 1998; Spacek and Harris, 2004). Moreover, spinules embedded within SBBs seemed to have a range of sizes and shapes, even within a single spinule origin category (e.g., postsynaptic spine spinules). Some spinules, particularly from select postsynaptic spines and adjacent axons, had thin initial invaginations into their SBB but then expanded into an anchor or hook-like shape (Figs. 5*A*, 6*B*, 7*D*). In addition, SBBs sometimes enveloped a portion of the head of their postsynaptic spine partners (Figs. 6*A*, 7*C*,*F*), a phenomenon previously described for TC boutons and their postsynaptic spines in mouse barrel cortex (Rodriguez-Moreno et al., 2018). Rarely, SBBs in older animals were observed enveloping spinules from multiple neurites (5.6% and 1.9% of SBBs for p60 and >p90, respectively; Figs. 4*A*, 5*E*,*F*, 6*C*). Thus, SBBs in L4 of V1 contain a diverse array of spinules from postsynaptic and adjacent neurites and glia, possessing a range of morphologies suggestive of distinct functions.

### Spinule prevalence across development: 3D FIBSEM analyses

**Figure 8.**
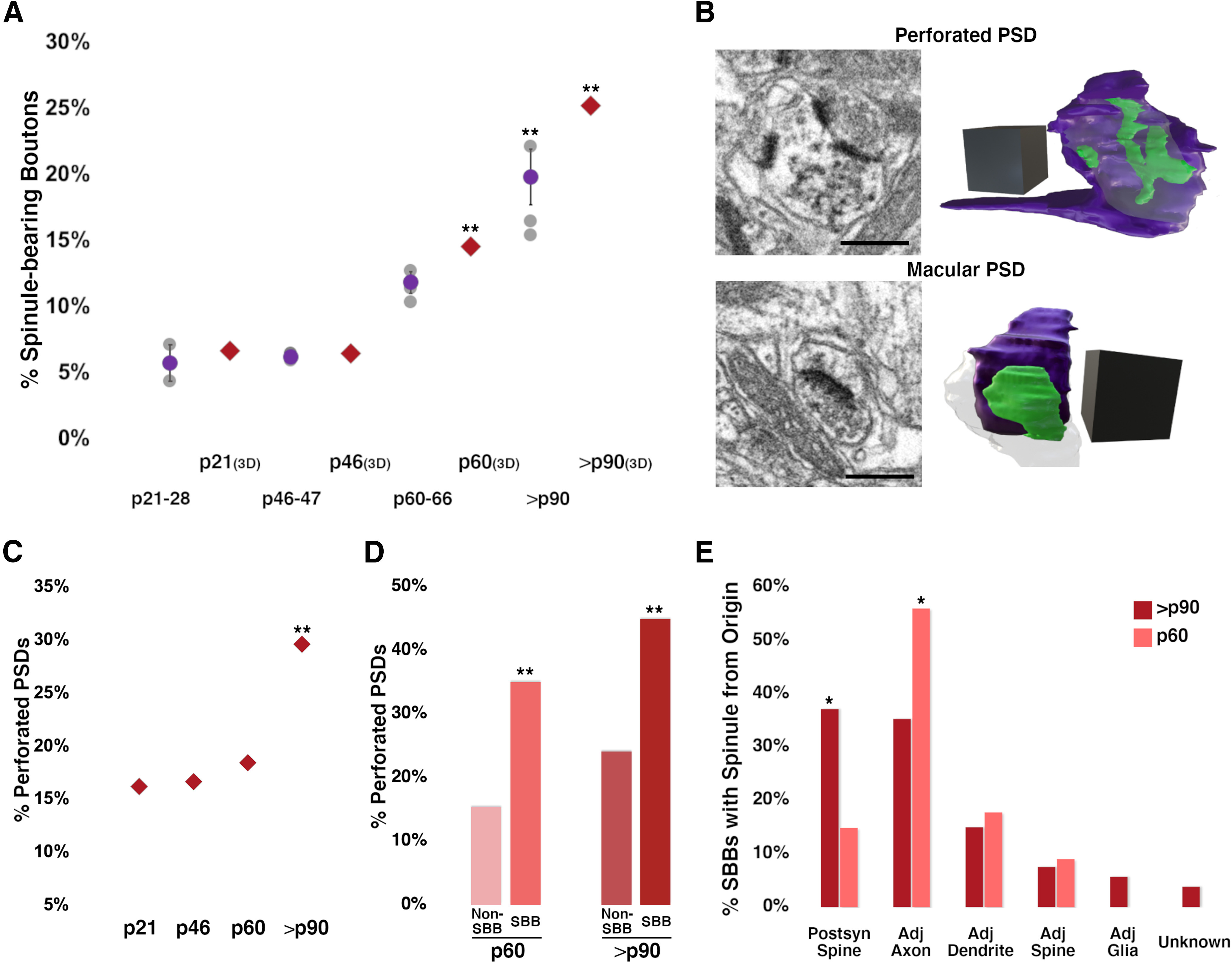
SBBs progressively engulf spinules as plasticity wanes in V1. ***A***, Percentages of excitatory SBBs across the developmental ages examined in 2D TEM and FIBSEM (3D) images. 3D FIBSEM data at p21, p46, p60, and >p90 are represented by red diamonds, 2D data as shown in Figure 2*D*. Statistical comparisons were performed between p21 and p46, p46 and p60, and p60 and >p90 (for details, see Table 3). ***B***, FIBSEM images showing an SBB with a synapse onto a postsynaptic spine containing a perforated PSD (top panel), and an SBB with a with a synapse onto a spine displaying a macular PSD (bottom panel). 3D reconstructions of each SBB and their corresponding PSDs are shown to the right. SBBs shown in purple, PSDs shown in green, postsynaptic spines are made transparent to visualize PSDs. Identical SBBs as shown in Figure 7*C*,*D*. ***C***, FIBSEM developmental analysis showing the average percentages of excitatory presynaptic boutons with perforated PSDs in L4. ***D***, FIBSEM analysis showing the percentages of SBBs versus Non-SBBs (i.e., excitatory presynaptic boutons without spinules) with perforated PSDs at p60 and >p90. ***E***, FIBSEM analysis showing the percentages of p60 and >p90 SBBs containing at least one spinule from a defined neurite or glial origin. For example, ∼56% of SBBs at p60 contain at least one spinule from an adjacent axon. Scale bars = 0.5 μm for FIBSEM images in ***B***; 3D scale cubes = 0.5 μm^3^ for FIBSEM reconstructions in ***B***; *p < 0.05, **p < 0.015.

Our quantitative 3D FIBSEM analyses of 676 excitatory boutons revealed that the percentage of SBBs within the synaptic bouton population of L4 remains at ∼6.5% from before eye-opening (p21) until the height of the critical period for plasticity (p46; Fig. 8*A*). Yet by p60, 14.5% of excitatory boutons contain spinules (a 127% increase from p46 levels), and by >p90 spinules are embedded within 24.2% of excitatory boutons (a 67% increase from p60 levels; Fig. 8*A*; Tables 2, 3). These data mirror the trends we observed in our 2D TEM analyses of spinule prevalence across these same developmental time points (Fig. 8*A*). Together, these analyses demonstrate that the rate and/or maintenance of spinule engulfment by excitatory boutons in L4 of V1 is most likely regulated by mechanisms that parallel the waning of cortical plasticity, suggesting spinules act to stabilize functionally and/or morphologically mature neocortical synapses.

**Table 2:** Percentages of SBBs, perforated PSDs, spinule origins, and sampled structures for FIBSEM analyses

FIBSEM										
Age	Total boutons	Total synapses	% SBBs	% Perforated PSDs	% PS spine	% Adj axon	% Adj spine	% Adj dendrite	% Adj glia	% Unknown
>p90	223	237	24.2	29.5	37.0	35.2	7.4	14.8	5.6	3.7
p60	235	256	14.5	18.4	14.7	55.9	8.8	17.7	0.0	0.0
p46	157	163	6.4	16.6	10.0	50.0	0.0	40.0	0.0	0.0
p21	61	62	6.6	16.1	25.0	25.0	0.0	25.0	0.0	25.0

Total sampled boutons and synapses for each age are listed under total boutons and total synapses columns for FIBSEM analyses at each age examined. Percentages of SBBs and perforated PSDs in FIBSEM analyses are listed in their respective columns for each age examined. Percentages of SBBs containing at least one spinule from a neurite/glial source are listed in their respective columns for each age examined. PS = postsynaptic; adj = adjacent (i.e., not synaptic); unknown = spinules whose origin could not be determined (for details, see Materials and Methods). Since some SBBs contained spinules from multiple sources, percentages of spinules from all origins may sum to greater than 100%.

**Table 3:** Statistical Comparisons for 2D Transmission Electron Microscopy (TEM) and 3D Focused Ion Beam Scanning Electron Microscopy (FIBSEM) Analyses.

**Comparison**		**Test**	**n (animals, total sampled structures)**	**% Change**	**Power** **(post-hoc, two-tailed)**	**p value**
**Synapse Length**						
All Ages	ANOVA, one-way					6.7 x 10^-7^*
>p90 vs. p60-66		Bonferroni post hoc t Test, two-tailed	>p90 (3, 323); p60-66 (3, 266)	16%	0.96	2.1 x 10^-4^*
p60-66 vs. p46-47		Bonferroni post hoc t Test, two-tailed	p60-66 (3, 266); p46-47 (2, 196)	5%	0.14	0.327
p46-47 vs. p21-28		Bonferroni post hoc t Test, two-tailed	p46-47 (2, 196); p21-28 (2, 152)	2%	0.07	0.669
**Bouton Area**						
All Ages	ANOVA, one-way					6.0 x 10^-13^*
>p90 vs. p60-66		Bonferroni post hoc t Test, two-tailed	>p90 (3, 319); p60-66 (3, 254)	20%	0.82	0.005*
p60-66 vs. p46-47		Bonferroni post hoc t Test, two-tailed	p60-66 (3, 254); p46-47 (2, 196)	39%	0.98	6.1 x 10^-5^*
p46-47 vs. p21-28		Bonferroni post hoc t Test, two-tailed	p46-47 (2, 196); p21-28 (2, 141)	93%	0.99	1.34 x 10^-11^*
**Spinule Area**						
All Ages	ANOVA, one-way					0.671
**Spinule-Bearing Bouton Area**						
>p90 SBBs vs. No Spinules		Mann-Whitney U test	>p90 SBBs (3, 57); >p90 NoSpin (3, 266)	88%	0.99	2.69 x 10^-9^*
**% Spinule-Bearing Boutons**						
**2D**						
All Ages (4 x 2 table)		X^2^ Test, two-tailed, Bonferroni corrected				5.3 x 10^-5^*
>p90 vs. p60-66		X^2^ Test, two-tailed, Bonferroni corrected	>p90 (3, 319); p60-66 (3, 254)	68%	0.73	0.009*
p60-66 vs. p46-47		X^2^ Test, two-tailed, Bonferroni corrected	p60-66 (3, 254); p46-47 (2, 196)	92%	0.52	0.041
p46-47 vs. p21		X^2^ Test, two-tailed, Bonferroni corrected	p46-47 (2, 196); p21-28 (2, 141)	8%	0.04	0.863
**FIBSEM**						
All Ages (4 x 2 table)						< 1.0 x 10^-5^*
>p90 vs. p60		X^2^ Test, two-tailed, Bonferroni corrected	>p90 (1, 223); p60 (1, 235)	67%	0.81	0.008*
p60 vs. p46		X^2^ Test, two-tailed, Bonferroni corrected	p60 (1, 235); p46 (1, 157)	127%	0.70	0.014*
p46 vs. p21		X^2^ Test, two-tailed, Bonferroni corrected	p46 (1, 157); p21 (1, 61)	3%	0.03	0.959
**% Perforated PSD**						
**FIBSEM: All Boutons**						
All Ages (4 x 2 table)		X^2^ Test, two-tailed, Bonferroni corrected				0.003*
>p90 vs. p60		X^2^ Test, two-tailed, Bonferroni corrected	>p90 (1, 237); p60 (1, 256)	61%	0.82	0.004*
p60 vs. p46		X^2^ Test, two-tailed, Bonferroni corrected	p60 (1, 256); p46 (1, 163)	11%	0.06	0.639
p46 vs. p21		X^2^ Test, two-tailed, Bonferroni corrected	p46 (1, 163); p21 (1, 62)	3%	0.04	0.937
**FIBSEM: SBBs vs. Non-SBBs**						
>p90 SBBs vs. Non-SBBs		X^2^ Test, two-tailed, Bonferroni corrected	>p90 SBBs (1,58); >p90 Non-SBBs (1,179)	87%	0.83	0.001*
p60 SBBs vs. Non-SBBs		X^2^ Test, two-tailed, Bonferroni corrected	p60 SBBs (1,40); p60 Non-SBBs (1,216)	129%	0.77	0.003*
**Spinule Origins: >p90 vs. p60-66**						
% Postsynaptic Spine (>p90 vs. p60)		X^2^ Test, two-tailed, Bonferroni corrected	>p90 (1, 54); p60 (1, 34)	152%	0.63	0.024*
% Adjacent Axon (>p90 vs. p60)		X^2^ Test, two-tailed, Bonferroni corrected	>p90 (1, 54); p60 (1, 34)	37%	0.48	0.030*
% Adjacent Dendrite (>p90 vs. p60)		X^2^ Test, two-tailed, Bonferroni corrected	>p90 (1, 54); p60 (1, 34)	16%	0.06	0.724
% Adjacent Spine (>p90 vs. p60)		X^2^ Test, two-tailed, Bonferroni corrected	>p90 (1, 54); p60 (1, 34)	6%	0.05	0.271
% Adjacent Glia (>p90 vs. p60)		X^2^ Test, two-tailed, Bonferroni corrected	>p90 (1, 54); p60 (1, 34)	n/a	0.24	0.161

Statistical tests used for 2D TEM and 3D FIBSEM analyses are listed, grouped by each quantified morphology category (left most column) and age group comparison (“Comparison” column). Percent change between means are calculated as: $\left|\frac{\left(Age_{2}-Age_{1}\right)}{Age_{1}}\right|x100$, where *Age_2_*= Mean of older age group, and *Age_1_*= Mean of younger age group. Power is the post-hoc two-tailed Fisher’s exact test achieved statistical power for each comparison, given the two proportions and sample sizes (n), with ∝ error probability = 0.05. * = comparisons where p < 0.05 or Bonferroni corrected value. Sample sizes for SBB vs. Non-SBB perforated PSDs reflect the total number of SBB and Non-SBB synapses for each age group, and ‘n’ for ‘Spinule Origins’ comparisons reflect the total number of spinule-bearing boutons (SBBs) within each group. PSDs = Postsynaptic densities. 2D = TEM analyses. Non-SBBs = excitatory presynaptic boutons without spinules.

### Perforated PSDs and SBB prevalence

As the prevalence of separations/perforations in PSDs have been suggested to correlate with levels of plasticity and spinule prevalence (Jones et al., 1991; Spacek and Harris, 2004), we sought to determine the developmental trajectory for the appearance of perforations in PSDs at excitatory synapses in our data. We reasoned that if spinules and perforated PSDs were both linked to heightened levels of plasticity and/or if spinules were responsible for producing perforations in PSDs as they invaginate into boutons, that spinule prevalence and levels of perforated PSDs would increase in parallel. However, we found that levels of perforated PSDs at excitatory synapses remained stable between p21–p60 (∼16–18%), followed by a significant increase to ∼30% by >p90 (Fig. 8*B*,*C*; Tables 2, 3). Furthermore, the percentage of perforated PSDs at synapses formed by SBBs did not appreciably change across the ages examined (50%, 40%, 35%, and 45% for p21, p46, p60, and >p90, respectively, χ^2^ test, 4 × 2 contingency table, *p* = 0.56), although our power to detect changes in perforated synapses at p21 and p46 SBBs is low given the small percentages of SBBs at these ages. Nevertheless, at p60 and p90 ages, SBBs have higher percentages of perforated PSDs versus presynaptic boutons without spinules (non-SBBs; 35.0% vs 15.3%, *p* = 0.003 at p60, 44.8% vs 24.0%, *p* = 0.001 at p90, % perforated PSDs for SBBs vs non-SBBs, respectively; Fig. 8*D*; Table 3). Together, these data demonstrate that perforated PSDs are present at ≤50% of SBBs across development, yet a positive relationship exists between spinule envelopment and PSD perforation within a subset of SBBs at p60 and p90.

### Origins of spinules within SBBs

Our FIBSEM analyses also allowed us to track the parent structure within the neuropil for every spinule embedded within developing SBBs in our image volumes from L4 of V1. We hypothesized that spinules from postsynaptic spines, particularly larger anchor-like spinules (Figs. 6*B*, 7*D*) embedded within SBBs might encourage the maintenance and stability of synaptic connections. In contrast, spinules from non-synaptic adjacent neurites (e.g., axon/boutons, dendrites) might enable novel forms of communication or neuronal circuit remodeling (Spacek and Harris, 2004; Petralia et al., 2018). We focused our analysis of spinule origins on p60 and >p90 ages where we had sufficient SBBs (see Table 3) to attain reasonable statistical power when comparing subsets of SBBs containing spinules from unique neurite/glial sources. We found that at both of these ages, SBBs primarily contained spinules from postsynaptic spines and adjacent axons/boutons, with a minority from other sources (Fig. 8*E*; Table 2). However, at p60, a majority of SBBs (56%) had spinules originating from adjacent axons, and only ∼15% of SBBs contained spinules from their postsynaptic spine partners. Yet by >p90, a significantly smaller percentage of SBBs contained spinules from adjacent axons (∼35%), while a significantly larger percentage of SBBs contained spinules from their postsynaptic spine partners (37%). These data indicate that SBBs in L4 of V1 increasingly engulf spinules from postsynaptic spines as they mature, potentially acting to facilitate the stability of these connections. Whereas, SBBs decrease their preference for spinules from adjacent axons by >p90, potentially signaling the decreasing importance of these spinules for enabling extrasynaptic communication and/or circuit remodeling at more mature synapses.

## Discussion

Despite decades of discussion within the TEM literature, the developmental prevalence and origins of spinules within cortical boutons has remained obscure. In this study, we performed 2D TEM and 3D FIBSEM analyses to determine the proportion of excitatory cortical boutons that envelop spinules across ferret postnatal development in V1 and quantified the percentages of spinules embedded within neocortical boutons that arise from unique neurite/glial sources. We found that (1) the proportion of SBBs within the L4 excitatory bouton population increases as physiological and morphological plasticity wanes, suggesting spinules play a role in stabilizing mature cortical synapses, (2) SBBs preferentially engulf spinules from postsynaptic spines and adjacent boutons/axons by p60 and into late adolescence, and (3) nearly one-quarter of the excitatory synaptic bouton population in L4 of late adolescent ferret V1 contain spinules.

### 2D TEM versus 3D FIBSEM considerations

In order to address interanimal variability and variability across the depth of L4 in our developmental analyses, we characterized cortical bouton developmental morphology and the percentages of cortical SBBs (*n* = 10 animals, 910 synaptic boutons) using 2D TEM, and then used some of the same exact tissue to perform new 3D FIBSEM analyses (*n* = 4 animals, 676 synaptic boutons). While we took great care to only sample each synapse once and to analyze each synaptic profile we encountered, there are limitations associated with 2D TEM analyses. Since a percentage of small synapses are undersampled in a 2D analysis (Colonnier and Beaulieu, 1985), we likely failed to include some smaller synapses in our 2D TEM analyses. While we cannot rule out this possibility, our 3D FIBSEM data provide strong confirmatory support for both the trends and relative differences in SBB prevalence across the ages we examined (Fig. 8*A*; Table 3). In addition, our 2D analyses were able to demonstrate the variability in SBB prevalence present across animals (e.g., 15–22% across three animals at >p90) and suggest that SBBs are larger than boutons without spinules (Extended Data Fig. 2-2), a testable prediction for future FIBSEM experiments. Moreover, our 2D data reveal the relatively large variability in spinule sizes within SBBs across animals at later developmental time points (Fig. 2*C*; Table 1), suggesting spinules display a range of morphologies that may be related to their origin (Fig. 8*E*).

Furthermore, to exclude bouton organelles (e.g., endoplasmic reticula, lysosomes, synaptic vesicles) from our SBB analyses within 2D ∼60 nm-thick TEM sections, we adopted conservative criteria (see Materials and Methods) to positively identify membrane bound structures as spinules within 2D bouton profiles. Accordingly, our 2D TEM analyses failed to include some number of small spinules across developmental ages because of their small size and/or our stringent quantitative criteria. Indeed, we found slightly higher SBB prevalence at p60 and >p90 in our FIBSEM analyses (14.5% and 24.2% SBBs, p60 and >p90, respectively) versus our 2D TEM analyses (11 ± 0.8% and 18 ± 2%, mean ± SEM, p60 and >p90, respectively) that are likely attributable to small spinules that were missed in our 2D analyses. Therefore, our FIBSEM analyses likely provide more accurate estimates of absolute SBB prevalence. However, the differences in percentages of SBB prevalence between our 2D TEM and FIBSEM analyses were not statistically significant (*p* = 0.07, *p* = 0.38, *p* = 0.95, and *p* = 0.88, for >p90 vs >p90, p60–p66 vs p60, p46–p47 vs p46, and p21–p28 vs p21, 2D TEM vs FIBSEM, respectively; χ^2^ test, two-tailed, Bonferroni corrected).

### Relationship of perforated synapses to synaptic spinules

Increases in the proportion of PSDs displaying perforations and “horseshoe” morphology are associated with increases in cortical activity (Calverley and Jones, 1990; Geinisman et al., 1993; Toni et al., 1999; Harris et al., 2003) and can occur concomitant with increased spinule emergence from dendritic spines (Sorra et al., 1998; Toni et al., 1999; Spacek and Harris, 2004). Accordingly, one hypothesis for the functional significance of perforated PSDs might be the necessity of PSDs to perforate to allow for spinule growth into a bouton. However, similar to our observations in L4 of V1, a majority of spinules from hippocampal spines seem to emerge from the edge or distant from the PSD rather than from the middle of the PSD (Harris and Stevens, 1989; Spacek and Harris, 2004), suggesting that spinule emergence is not responsible for perforating most PSDs. Similarly, we found that increases in SBB prevalence preceded the significant developmental increase in perforated PSDs (Fig. 8*A–C*) and that only 35–50% of SBBs in V1 had synaptic partners with perforated PSDs, arguing against a causal relationship between spinule protrusions and perforated PSDs. These data are in line with multiple reports from rat neocortex where spinules have only been reported to appear within ∼19% (Bozhilova-Pastirova and Ovtscharoff, 1999; rat S1), ∼17% (Spacek, 1985; rat V1), and 13–37% (Jones and Calverley, 1991; rat parietal cortex across lifespan) of synapses with perforated PSDs. Hence, while our data demonstrate that spinules are increasingly engulfed by neocortical excitatory synaptic boutons over development, and the percentage of neocortical PSDs displaying perforations seems to increase as an animal matures (Geinisman et al., 1987; Calverley and Jones, 1990; Jones and Calverley, 1991; Dufour et al., 2016), it seems unlikely that spinule emergence or engulfment by SBBs are causally related to PSD perforations at all SBB-containing synapses, at least in ferret V1. However, at p60 and p90, SBBs contain nearly twice the number of perforated PSDs as do boutons without spinules (Fig. 8*D*; Table 3), suggesting that spinule envelopment and PSD perforations are mechanistically related in subsets of SBBs. Indeed, a recent live-imaging study of cortical neurons in culture found that dendritic spines protruding large, stable “long-lived” spinules were more frequently associated with perforated PSDs (78%) than were spines projecting short-lived spinules (23%; Zaccard et al., 2020). Thus, it may be that SBBs containing larger, more stable spinules are able to influence the dynamic partitioning of PSDs. However, whether the relationship between larger spinules within SBBs and perforated PSDs is maintained across development in vivo remains an open question.

### Nearly one-quarter of excitatory cortical boutons contain spinules

We found that nearly one-quarter (24.2%) of excitatory boutons in L4 of late adolescent (>p90) ferret V1 contain spinules. To our knowledge, there have only been two other published reports that investigated the proportion of excitatory bouton populations in vivo that contain spinules. Erisir and Dreusicke (2005) found that in L4 of ferret V1, 10% of anterogradely-labeled TC boutons at p35–p49, and 28% of labeled TC boutons in adult contained spinules. Since, similar to our measurements of SBBs, this study found that labeled TC boutons are larger than their unlabeled counterparts, it is tempting to speculate that the SBBs that we sampled may be largely of TC origin. Indeed, a FIBSEM and serial TEM study of anterogradely labeled TC boutons in p60–p65 mouse barrel cortex found that 40% of TC axospinous synapses had spinules or spine heads protruding into TC boutons (Rodriguez-Moreno et al., 2018). However, since spinules were not a focus of either of these TC studies, the variety, proportions, and specificity of spinule types for TC boutons versus non-TC intracortical boutons remain unclear. Moreover, L4 TC boutons only represent ∼5–20% of the total excitatory bouton population in L4 of mammalian V1 (Ahmed et al., 1994; Peters et al., 1994; Latawiec et al., 2000; da Costa and Martin, 2009; Bopp et al., 2017; Garcia-Marin et al., 2019). Hence, given that 24.2% of excitatory boutons at >p90 were SBBs, it seems likely that at least a portion of the SBBs we encountered were intracortical excitatory boutons.

Examining SBBs within the TC population (e.g., VGluT2-containing) and excitatory intracortical bouton population (e.g., VGluT1-containing) are logical next steps in determining whether spinules from specific origins are preferentially engulfed by these functionally disparate classes of boutons. However, regardless of whether spinules are engulfed by specific bouton subtypes, our data suggest that over a thousand synapses per individual L4 cortical neuron (DeFelipe and Fariñas, 1992) contain SBBs. As such, spinules are poised to play prominent roles in facilitating synaptic stability (i.e., in SBBs physically connected to their postsynaptic spines), and potentially augmenting neuronal communication through transendocytosis (Spacek and Harris, 2004) and/or ephaptic coupling (Wagner and Djamgoz, 1993). Thus, an understanding of how spinules affect synapse function and/or stability will significantly impact our insight into neuronal input-output relationships.

### Spinule origins and potential functions

To our knowledge, these data represent the first investigation into the origins of spinules within neocortical presynaptic boutons. A pioneering study into the 3D structure of spinules within CA1 hippocampus found that out of 254 identified spinules, a majority emerge from dendritic spines (∼86%), and a minority emerge from axons (∼12%) and dendritic shafts (∼1%; Spacek and Harris, 2004). Furthermore, these hippocampal spinules are engulfed by a majority of presynaptic boutons (∼90%). The relative relationships between the proportions of spinule-projecting objects in hippocampus are in rough agreement with our data demonstrating that cortical boutons in L4 of late adolescent ferret engulf a majority of dendritic spines (44%: 37% postsynaptic spines and ∼7% adjacent non-synaptic spines) and boutons/axons (35%). However, if SBBs comprise the majority of spinule-engulfing structures in primary sensory neocortex as they seem to do in the hippocampus, there may be interesting systematic differences in the proportions of spinule projecting objects and/or SBB preference for specific spinule types between the hippocampus and neocortex. For example, axons/bouton spinules appear to be overrepresented within >p90 cortical SBBs (∼35%) in comparison to the proportion of these spinules that emerge from boutons/axons in rat CA1 (∼12%). Thus, it will be interesting to investigate the origins of spinules within SBBs in CA1, as well as to determine whether activity (e.g., LTP) differentially influences the types of neurites embedded within hippocampal SBBs.

Largely because of evidence demonstrating that increases in electrical and chemical LTP protocols are able to transiently increase the emergence of spinules from dendritic spines (Schuster et al., 1990; Geinisman et al., 1994; Toni et al., 1999; Harris et al., 2003; Ueda and Hayashi, 2013), it has been suggested that spinules function as circuit remodeling and signaling elements and/or as a mechanism to retrieve presynaptic membrane during periods of heightened activity (Spacek and Harris, 2004; Tao-Cheng et al., 2009). While the present study did not directly address spinule function or manipulate activity state, we have demonstrated that cortical boutons progressively engulf spinules over postnatal maturation, but not following the onset of sensory activity or during a period of heightened plasticity in cortex. While it is possible that transient increases in spinule emergence occur immediately following eye-opening and during heightened states of plasticity in cortex, excitatory synaptic boutons do not appear to engulf higher percentages of spinules during these developmental time points. Indeed, if spinules enhance or augment synaptic communication, presynaptic membrane retrieval, or help to maintain synaptic strength/stability, these functions likely depend on spinules first becoming enveloped by presynaptic boutons. Thus, in light of the current developmental and activity-dependent data on spinule emergence and engulfment, we argue for a model of spinule function wherein spinules: (1) act to progressively anchor presynaptic and postsynaptic elements together over development, potentially ensuring the stability and strength of key synapses within a functional circuit; and (2) enable circuit remodeling and/or membrane retrieval during brief periods of heightened activity.

In addition, it seems likely that the dramatic increase in membrane interface between boutons and spinules within SBBs (Calverley and Jones, 1990; Jones and Calverley, 1991; Rodriguez-Moreno et al., 2018) allows for some form of neuronal communication. For example, following increased activity in hippocampus, a majority of the tips of spinules from postsynaptic spines are coated in clathrin, and these clathrin-coated tips may pinch-off into their enveloping boutons (Spacek and Harris, 2004; Tao-Cheng et al., 2009). Furthermore, the large spinule-bouton membrane interface at synapses might allow for neuronal communication through ephaptic coupling, wherein a spinule that is tightly bound (i.e., high extracellular impedance) to its presynaptic bouton receives current flow during a presynaptic action potential (Wagner and Djamgoz, 1993; Faber and Pereda, 2018). If these subcellular processes take place, our data on spinule origins suggest that this form of ephaptic coupling would allow for unique forms of communication (e.g., non-synaptic axoaxonic communication) in L4 of V1. Nevertheless, it seems likely that spinules from specific sources (e.g., postsynaptic spines vs adjacent axons/boutons), and even spinules of different sizes from a single neurite source (Zaccard et al., 2020), will have distinct functional relationships with SBBs. Our data identify spinules from postsynaptic dendritic spines and adjacent axons as prime candidates for investigations into the functional consequences of spinule engulfment by SBBs in the neocortex.

In sum, spinules are conserved structural features of synapses that are embedded within nearly one-quarter of late adolescent excitatory boutons in L4 of V1. Spinules are progressively engulfed by presynaptic boutons as physiological and morphological measures of plasticity wane, suggesting that they have a role in the stabilizing and strengthening a select set of cortical synapses. Determining whether specific subsets of cortical excitatory and inhibitory (Tao-Cheng et al., 2009) boutons preferentially engulf spinules, as well as further defining the mechanisms for spinule induction, expression (Zaccard et al., 2020), maintenance (Ueda and Hayashi, 2013), engulfment, and potential communication, will be crucial to elucidating the role that synaptic spinules play within neuronal microcircuits.

## Synthesis

Reviewing Editor: Robert Kalb, Northwestern University Decisions are customarily a result of the Reviewing Editor and the peer reviewers coming together and discussing their recommendations until a consensus is reached. When revisions are invited, a fact-based synthesis statement explaining their decision and outlining what is needed to prepare a revision will be listed below. The following reviewer(s) agreed to reveal their identity: Joachim Lübke.

Both reviewers find great merit in this work. There are some minor issues to address that I, as REviewing Editor, can vet

## Author Response

RE: Manuscript en-NWR-0426-19R1

Title: Cortical Presynaptic Boutons Progressively Engulf Spinules as They Mature

Point-by-Point Author Response

We thank the reviewers for their detailed and constructive critique of our revised manuscript. Below, please find our point-by-point response (in blue font) to each of the remaining reviewer concerns and suggestions.

Reviewer #1

The authors have mostly addressed my initial concerns and comments.

My main point of criticism remains that, given the efforts in neuroanatomy to develop stereological methods to correct for sampling biases in brain tissue, I still believe the method of counting objects on 2D sections without any correction falls short of the state-of-the-art. In fact, many of the papers referenced by the authors address exactly this issue and/or use stereological methods that were developed for obtaining unbiased estimates (here are just the first four such papers from the reference list: Bopp et a. (2017), Colonnier and Beaulieu (1985), da Costa and Martin (2009), DeFelipe et al. (1999), ...).

However, in addition to this 2D sampling, the authors have now included FIBSEM analyses for all age groups. Even though absolute numbers differ somewhat between these methods, the basic trends appear to be similar. The absolute numbers that come from the FIBSEM analyses can be trusted more and should prove useful to other groups. Furthermore, the authors have now included a relatively thorough methodological discussion, pointing out caveats (and advantages) of both methods.

Overall, my recommendation is to accept this manuscript. However, there remain a few minor concerns that the authors should first address.

1. The authors do a good job of pointing out and discussing the bias in 2D sampling and biases that were introduced because different criteria for spinule identification had to be used between 2D and 3D EM. However, I feel the text should be less vague and evasive when it comes to these biases.

The authors collected impressively large datasets using both methods and sampling biases must necessarily be reflected in absolute numbers. I think it would actually strengthen the paper if the authors would be less vague in their wording and I would like to suggest following changes to the text:

- line 213, please leave out ‘may have’ or write something along the lines of ‘Because some smaller spinules are missed with 2D sampling...’.

The 2D analysis definitely excluded some smaller spinules that would have been counted in 3D FIBSEM, which becomes clear from figure 8A.

Corrected, now reads, “Therefore, our 2D analyses excluded some number of smaller spinules and consequently yielded reduced spinule prevalence”.

- please leave out ‘potentially’ biased in line 338. Sampling from 2D sections is actually biased.

Corrected, now reads, “However, while they have higher lateral resolution, 2D TEM analyses are biased toward omitting smaller objects...”

- line 458, please replace ‘may be’ with something like ‘since small structures are undersampled’.

Corrected, now reads, “Since a percentage of small synapses are undersampled in a 2D analysis...”

- line 470: Again, I think it would be good if the authors would acknowledge this bias more openly. It is not just ‘conceivable’ or possible that such a bias exists, but it must be reflected in the absolute numbers across their impressively large datasets.

Corrected, now reads, “Accordingly, our 2D TEM analyses failed to include some number of small spinules across developmental ages due to their small size and/or our stringent quantitative criteria.”

- line 472 - 476 This is an important point: It should be reported here in the text if the differences in % SBB between 2D and 3D EM are actually statistically significant. The authors say they found ‘slightly higher SBB prevalence [] in [] FIBSEM analyses...’. It would be important for the reader to know more about the statistics. This is an opportunity to assess the magnitude of bias between methods and would make the data much more interpretable.

Thank you for the suggestion. We now explicitly state that, “...the differences in percentages of SBB prevalence between 2D TEM and FIBSEM analyses were not statistically significant. Specifically, p = 0.07, 0.38, 0.95, and 0.88, for >p90 vs. >p90, p60-66 vs. p60, p46-47 vs. p46, and p21-28 vs. p21, 2D TEM vs. FIBSEM, respectively; X2 Test, two-tailed, Bonferroni corrected.

- I agree with the authors that the 2D and 3D sampling show the same trends. They might want to point out again at a prominent location in the discussion that if other groups would like to use their data, then the 3D FIBSEM data should be trusted more in terms of absolute numbers.

This is now noted immediately preceding the statement in line 472-476. Text now reads, “Therefore, our FIBSEM analyses likely provide more accurate estimates of absolute SBB prevalence.”

2. The clarity of the text has improved significantly overall, but here are some more sentences I found hard to parse:

Line 82 - 83: “...TC boutons in layer 4 (L4) of primary visual cortex (V1) contained spinule-like (i.e. putative spinules) 2D cross-sections...” What confuses me is that ‘a bouton contains a cross-section’, which I think is technically incorrect. I think what the authors are trying to say is that 28% of TC boutons contained spinules in cross-sections of V1 L4 tissue?

Our intention here is to describe the 2D section through a 3D bouton and/or spinule, as visualized in a 2D TEM image. While the term “cross-section” might more commonly refer to a perpendicular section through a 3D object, we (and others) use this term to describe the observed 2D section through a 3D object that has been cut at any apparent angle (e.g. oblique/tangential, perpendicular) to its Feret (i.e. longest) diameter. However, to clarify this statement, we have edited it to read, “...contained 2D spinule-like (i.e. putative spinule) profiles...”

Line 276: similar: I find the term ‘2D synapse length cross sections’ unclear; just ‘2D synapse length’ would be clearer?

We have now either omitted the term ‘cross sections’ or replaced this term with ‘profiles’ or ‘2D profiles’ (except where explicitly defined as ‘profiles’) throughout the manuscript to aid in clarity.

Line 532: ‘hundreds of thousands of synapses on individual L4 cortical neurons’ This sounds like each individual neuron would form synapses with hundreds of thousands of presynaptic partners...

What the authors may mean is that hundreds of thousands of synapses in the neuropil of L4 in V1 contain spinules?

We believe this may have been misread. The text on Line 532 read, “hundreds to thousands of synapses on individual L4 cortical neurons...”, meaning that between hundreds to thousands of synapses (depending on neuronal subtype and cortical layer/area) contain SBBs. We now clarify this text to more simply read, “our data suggest that over a thousand synapses per individual L4 cortical neuron (DeFelipe and Fariñas, 1992) contain SBBs.”

3. 2D EM is used to quantify the variability in % SBB across animals and cortical depth (line 333).

- Do the authors mean different cortical layers, which is how I understand cortical depth, or do they mean how far down in the tissue a sample is taken (e.g. within L4)?

- Even though (some of) these data are in the tables, I feel that the analyses on variability and cortical depth should be discussed in a bit more detail in the Results and Discussion - in particular because the authors use these analyses partly as the motivation behind the 2D EM.

We now include more discussion of this measured variability. Text now reads, “In addition, our 2D analyses were able to demonstrate the variability in SBB prevalence present across animals (e.g. 15 -22% across 3 animals at >p90) and suggest that SBBs are larger than boutons without spinules (Fig. 2-2), a testable prediction for future FIBSEM experiments. Moreover, our 2D data reveal the relatively large variability in spinule sizes within SBBs across animals at later developmental time points (Fig. 2C, Table 1), suggesting spinules display a range of morphologies that may be related to their origin (Fig. 8E).”

4. I did search, but I could not find any table legends, which led to some confusion on my part. If I understand the tables correctly, table 1 shows absolute measurements for 2D TEM, table 2; same for FIBSEM; table 3, % differences? Correct?

eNeuro asks for a title and legend for each table (on their website under ‘Preparing a Manuscript’) and it would really help to clarify what is shown.

We apologize for this oversight, the Table Legends that were included as separate files are now also included with the Figure Legends at the end of the manuscript document.

5. Ephaptic coupling is interesting and it would be nice to write one or two more sentences explaining it in a bit more detail.

We agree, we find the potential for ephaptic coupling at neocortical spinule to SBB appositions compelling. We describe this idea in the discussion by stating, “Furthermore, the large spinule-bouton membrane interface at synapses might allow for neuronal communication through ephaptic coupling, wherein a spinule that is tightly bound (i.e. high extracellular impedance) to its presynaptic bouton receives current flow during a presynaptic action potential (Wagner and Djamgoz, 1993; Faber and Pereda, 2018). If these subcellular processes take place, our data on spinule origins suggest that this form of ephaptic coupling would allow for unique forms of communication (e.g. non-synaptic axoaxonic communication) in L4 of V1.” Since we do not yet have data directly measuring extracellular space, impedance, or extracellular potentials at SBBs, we feel that describing where and how this form of communication might take place in more detail is premature.

6. The authors found that only 16 - 30% of all boutons in L4 have perforated PSDs - while up to 50% of the SBB have perforated PSDs. So it seems that perforated PSDs could appear preferentially in SBB. What fraction of the non-SBB have perforated PSDs? Is there a statistical difference between % of SBB that have perforated PSDs and the % of non-SBB that have perforated PSDs? I think this is interesting because the authors aim to link SBB prevalence and synaptic plasticity. It wouldn’t hurt to show such a comparison in a figure.

Thank you for the suggestion. We have included a statistical and graphical comparison in the percentages of perforated PSDs between SBB and Non-SBBs at p60 and p90 ages. We restricted the comparisons to these ages because of the relatively low total numbers of SBBs (and thus low statistical power) at p21 and p46. The statistical comparison (added to Table 3) indicates that SBBs have higher percentages of perforated PSDs at both p60 and p90 (35.0 vs. 15.3 % at p60, and 44.8 vs. 24.0 % at p90, p = 0.003 (p60) and 0.001 (p90), SBBs vs. Non-SBBs, respectively). We have added this comparison as Fig. 8D, and included it in Table 3, as well as the Results and Discussion sections.

The following two points (7 & 8) are suggestions, and given the amount of work and time the authors have already invested in revising the manuscript, it should be up to them if they want to follow up on them:

7. Some boutons form multiple synapses, and in mouse S1, these tend to be of thalamic origin (e.g. Bopp et a. (2017), Rodriguez-Moreno et al. (2018)). Did the authors look at the prevalence of SBB amongst these multisynaptic boutons? I think this may be interesting and could really add to their story, in particular because the authors discuss the potential relationship between TC synapses and spinules. Also, it may help address the concern of Referee 2 that the functional role of spinules should be addressed in more detail.

We agree that this is an interesting comparison. However, we found that there was not a significantly higher number of multisynaptic SBBs versus non-SBBs (p= 0.054 (p60) and p=0.69 (>p90), X2 Test, Bonferroni corrected) in L4 of V1.

8. I agree that a PSD has a volume if you take its thickness into account. If the authors wish to do so, I have no objections. However, this should be clarified and the rationale should be given. My own initial response probably reflects that many readers would easily be confused or surprised.

We have omitted reference to a PSD volume to aid in clarity.

Reviewer #2 The paper has now very much improved in all parts, in particular the results and discussion chapter. The manuscript is accompanied by good and informative illustrations. The nice touch is the comparison of data obtained with TEM and FIB-SEM. May the reviewer are allowed to make a comment in the use of both EM techniques. I think the authors are aware that both TEM and FIB-SEM have advantages and disadvantages. The most important one in my opinion also using both technologies is the difference in resolution when it comes to high-resolution where TEM images provide a much better image quality (compare Figure 1 with Figures 5, 6 this paper). In this respect the reviewer would like to address a question concerning the identification of perforated PSDs. It would be helpful to describe criteria on how perforated PSDs were defined and identified in their FIB-SEM analysis which are missing in the manuscript. Secondly, could the authors provide an FIB-SEM image at higher resolution for a perforated vs. a macular, non-perforated PSD which would be helpful for the reader.

There are still some grammatical errors throughout the manuscript.

Thank you. We now include a description of how perforated PSDs were defined and identified in our FIBSEM analyses in the Methods section. This text now reads, “PSDs were deemed ‘perforated’ if they displayed one or more separations in the electron-dense region of the PSD, whereas macular PSDs were defined as PSDs bereft of these separations (Fig. 8B). PSD separations needed to be seen in the same x/y location in at least two consecutive serial FIBSEM images (i.e. 8 nm in the z dimension) to count as perforations.” In addition, we have now included zoomed-in FIBSEM images and 3D reconstruction examples and of a perforated and a macular PSD as Fig. 8B. We have edited the manuscript and corrected grammatical errors.
