# Supplementary figures and images for: Cortical Presynaptic Boutons Progressively Engulf Spinules as They Mature
Source: eNeuro. 2020 Oct 14;7(5):ENEURO.0426-19.2020. doi: 10.1523/ENEURO.0426-19.2020 (PMC7568603; doi:10.1523/ENEURO.0426-19.2020)

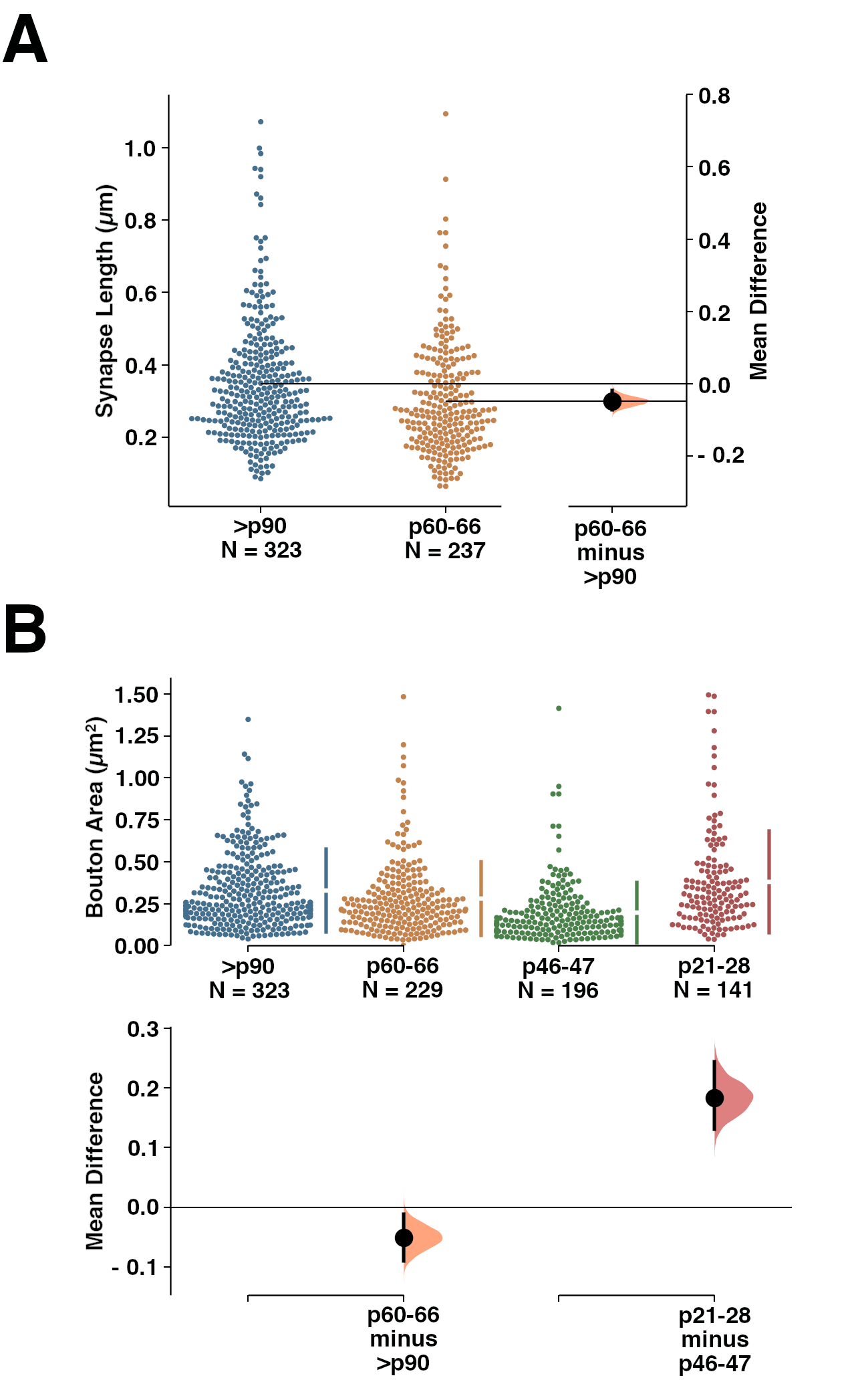

Supplement: Extended Data Figure 2-1 — Estimation statistics plots showing mean differences between developmental synapse length and bouton area comparisons. A, Gardner–Altman estimation plot (Gardner and Altman, 1986) showing the raw synapse profile length values for >p90 and p60–p66 groups (left y-axis), as well as the mean difference (black dot near right y-axis), and the bootstrap 95% confidence interval (vertical error bar from black dot). Mann–Whitney U test, p = 1.5 × 10^−4^. B, Estimation plot showing raw excitatory bouton areas for all developmental age groups examined. Plots marked as in A, except that mean differences between groups and confidence intervals are shown below each comparison. Mann–Whitney U test, p = 0.004 (p60–p66 vs >p90), and 2.8 × 10^−14^ (p21–p28 vs p46–p47). Download Figure 2-1, TIF file. [file enu-eN-NWR-0426-19-s02.tif]

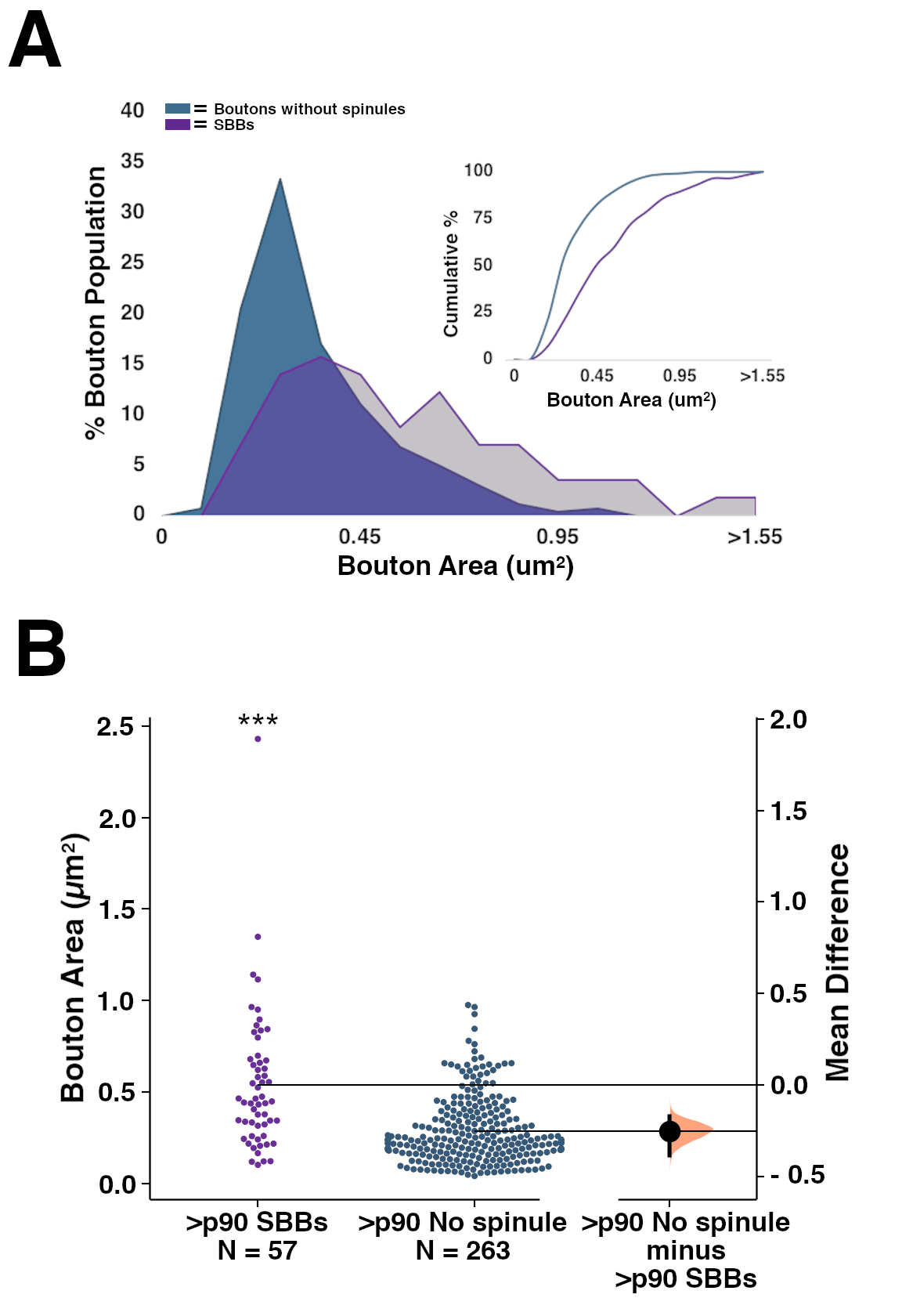

Supplement: Extended Data Figure 2-2 — SBBs are larger than boutons without spinules (no spinule). A, Histogram showing the distribution for the percentage of >p90 SBBs (purple) and >p90 presynaptic boutons without spinules (blue) with various sized areas. Inset, Cumulative histogram of this same comparison. B, Estimation plot showing the raw data, mean difference (black dot), and bootstrapped 95% confidence interval (lines extending from dot) for areas from >p90 SBBs and boutons without spinules. Mann–Whitney U test, p = 2.7 × 10^−9^; ***p < 0.001. Download Figure 2-2, TIF file. [file enu-eN-NWR-0426-19-s03.tif]
